# Re‐Interpreting Genetic Offset: Quantifying the Least Required Evolutionary Rate Under Climate Change at the Mediterranean Range Margin of European Beech

**DOI:** 10.1111/eva.70230

**Published:** 2026-04-08

**Authors:** Josep Morando‐Milà, Oriol Grau, Bartosz Ulaszewski, Albert Vilà‐Cabrera, Josep Peñuelas, Alistair Jump, Ivan Scotti

**Affiliations:** ^1^ Facultat de Biologia Universitat de Barcelona Barcelona Catalonia Spain; ^2^ INRAE URFM Avignon France; ^3^ CREAF Cerdanyola del Vallès Catalonia Spain; ^4^ Global Ecology Unit CREAF‐CSIC‐UAB CSIC Bellaterra Catalonia Spain; ^5^ Parc Natural de l'Alt Pirineu Llavorsí Catalonia Spain; ^6^ Wydział Nauk Biologicznych Uniwersytet Kazimierza Wielkiego w Bydgoszczy Bydgoszcz Poland; ^7^ Biosciences Department University of Vic – Central University of Catalonia Vic Catalonia Spain; ^8^ School of Life and Environmental Sciences University of Stirling Stirling Scotland UK

**Keywords:** climate change, *Fagus sylvatica*, genetic offset, local adaptation, population genomics, REvoRate

## Abstract

European beech ( *Fagus sylvatica* L.) spans a wide range of European climates and exhibits evidence of local adaptation, which supports its persistence under diverse conditions. We analysed 18 populations—distributed across an altitude gradient on the southwestern geographic range edge of the species—using landscape genomics to assess their adaptive variation and vulnerability to future climatic conditions. We uncovered weak but structured genetic differentiation, revealing three main climate‐tied genetic groups. Combining multiple Genotype–Environment Association (GEA) approaches—linear, such as Latent Factor Mixed Models (LFMM) or Redundancy Analyses (RDA), and non‐linear (Gradient Forest)—we identified 373 Single Nucleotide Polymorphisms (SNPs) detected by all GEA methods as being putatively associated with climate gradients. Using the Gradient Forest model, we mapped genetic offset across all 21st century periods under a key climate scenario: Shared Socioeconomic Pathway (SSP) 5, forcing 8.5 W/m^2^ (SSP585) and across all SSPs for 2061–2080, identifying Pyrenean and pre‐Pyrenean regions as maladaptation hotspots. To capture temporal dynamics, we introduce a novel approach to interpret genetic offset. The Required Evolutionary Rate (REvoRate) quantifies the minimum genetic change per year needed to keep pace with projected climates. Joint interpretation of offset and REvoRate revealed that some stands with moderate offsets face high short‐term adaptive demands, while others with larger offsets are required to evolve more gradually. The drought‐temperature gradient emerged as the main driver of allele frequency turnover, with geography contributing through isolation by distance. Together, genetic offset and REvoRate provide a dynamic framework to assess temporal maladaptation risk. Our results highlight the need to integrate standing genetic variation and evolutionary potential into forest management and conservation planning to ensure the persistence of *F. sylvatica* in one of its most climate‐vulnerable range margins.

## 1 Introduction

Climate is a key driver of species distributions and habitat availability (Araújo and Luoto 2007; Woodward and Williams 1987). In particular, vegetation patterns are closely tied to environmental conditions. However, climate is rapidly changing, trending towards a warmer and drier context in many regions (Calvin et al. 2023) and increasing the frequency, magnitude and duration of extreme events such as droughts (Goodess 2013). These shifts are already having adverse effects on European forests (Bonannella et al. 2024), where the persistence of tree species increasingly depends on their ability to tolerate warming temperatures and reduced precipitation (Vanhove et al. 2021).

The climate envelope of a species often encompasses diverse environmental gradients, which impose varying selective pressures on populations across space and time. As a result, natural selection can promote genetic variants that are better suited to local conditions in a process known as local adaptation. This phenomenon has been documented in many taxa, including plants (Cork Oak in Vanhove et al. 2021) and animals (North American grey wolves in Schweizer et al. 2016).

Understanding the genetic basis of local adaptation is essential for anticipating species' responses to climate change (Savolainen et al. 2013). Trees are long‐lived organisms– *Fagus sylvatica* L. (European beech), the focus of this study, often exceeds 300 years of age and does not reach sexual maturity until 40–50 years of age (Oliet et al. 2012). These life‐history traits may constrain the speed of adaptation, where environmental changes may be too fast for Darwinian selection to cope with. Moreover, the long juvenile phase makes it difficult to experimentally test population fitness under different climate scenarios (Lazic et al. 2024). Besides, Jump, Hunt, Martínez‐Izquierdo, and Peñuelas (2006) have shown that the genetic composition of age cohorts can be driven by the climate experienced at establishment, thus suggesting that populations as a whole can be composed of uneven‐aged groups selected by changing environmental conditions.

At the same time, trees are capable of long‐distance gene flow, allowing the exposure of original genetic combinations to novel environments, thus promoting adaptive evolution. Still, it remains unclear whether this could compensate for the long‐generation times (Kremer et al. 2012). If selection is not strong enough, this phenomenon can reduce the levels of local adaptation for the population receiving the alleles via gene flow (Tigano and Friesen 2016). In contrast, when the pressures are sufficient to promote adaptive divergence processes, these take place over short time spans thanks to standing genetic variation that supports local microgeographic divergence processes, which can maintain genetic diversity at the landscape level (Modica et al. 2024) and fuel rapid evolution processes and adaptations to new conditions at the genomic level (Saleh et al. 2022).

To predict whether these forests will be at risk of maladaptation in the future due to environmental shifts, there must first be local adaptation. Genotype–Environment Association (GEA) analyses provide a way to identify which parts of the genome show allele frequencies correlated with environmental variables (Duruz et al. 2019; Rellstab et al. 2015; Selmoni et al. 2021), and consequently allow us to identify genomic regions that are putatively involved in climate adaptation under the postulate that the genetic composition of the population is currently optimally adapted to their environment.

European beech diverged from its sister species during the Quaternary. After the Last Glacial Maximum, beech started dispersing from the refugia where it had survived (Magri 2008). These were very fragmented and predominantly located in ice‐free areas in southern latitudes. Many macrofossils have been found in Catalonia, dating from 13 to 10 kyr 14C BP (15.4–11.5 kyr Cal BP), even though the expansion in the mountains in the north of the Iberian Peninsula is more recent, having occurred 4–3 kyr 14C (4.5–3.2 cal. kyr. BP) ago (Magri et al. 2006).

The decline of beech populations in the Mediterranean was observed over the past two millennia (Magri 2008). This phenomenon is attributed to the aridification of the climate, documented by pollen analysis (Jalut et al. 2000) and probably the increasing human impact (Magri 2008). Also, the elongation of summer and the decrease in precipitation caused by anthropogenic climate change have been described to hinder the seedling bank of beech forests in the sub‐Mediterranean (Robson et al. 2009). The region we have studied, NE Iberian Peninsula, is in the Mediterranean Basin (except for a small area, located in the Atlantic Basin). Impacts of climate change will be particularly relevant in this area through a decrease in precipitation and an increase in temperature (Blanco‐Pastor et al. 2021; Pauls et al. 2013; Vanhove et al. 2021), making it a good candidate to study how genomic composition might have to respond to these shifts.

Here, we studied the populations of *F. sylvatica* found in Catalonia to infer whether they show signals of local adaptation, surviving in one of its most southern distributions. We searched for the presence of loci under selection that covary with the local environmental conditions and, from these results, assess the genetic turnover across the landscape and predicted genetic maladaptation (Fitzpatrick and Keller 2015) for the regional distribution of beech, which is quantified by how far from the genetic‐climate optimum each population will be (Lazic et al. 2024; Vanhove et al. 2021). We used diverse GEA methods to assess the (linear or non‐linear) correlations between environmental variables and reference allele frequencies for a large set of Single Nucleotide Polymorphisms (SNPs) and used the output of non‐linear regressions to predict the genetic composition for all sites included in the potential distribution of *F. sylvatica* in Catalonia. This, in turn, provides the means to estimate the allelic turnover between extant populations, as well as the allelic turnover required for a population to adapt to future climate conditions, that is, genetic offset (Fitzpatrick and Keller 2015).

We present a novel interpretation of genetic offset, which allows us to assess not only how far the genetic composition is from the predicted optimum, but also which populations are at a greater risk of early maladaptation. This measure relates offset to time, allowing us to predict the Required Evolutionary Rate (REvoRate, expressed in units of genetic change per year) to complement the absolute offset (see Section 2 for more details on the computation). If a population shows a high offset and high REvoRate, this being the worst‐case scenario, it means that it is under high adaptive stress; if the offset is high but the rate is low, this indicates that there is a large, accumulated mismatch, but slow climate velocity (chronic lag); if the offset is low and the rate is high, it is an indicator of a rapid environmental change but small current mismatch (transient stress), and evidently, if both are low, the population is near the equilibrium. We suggest that using the two indices can lead to a finer interpretation of genetic offset values.

The analyses were carried out on genetic loci obtained from full resequencing of 18 wild stands, and mapped onto the reference genome for *F. sylvatica* (Mishra et al. 2022) named Bhaga, the annotation of which was used as a basis for enrichment analysis and to obtain a list of genes that play a crucial role in the adaptation of beech in Catalonia.

Seeing these forests through their genetics will give more insights into orienting conservation efforts and whether a provenance study and assisted gene flow are needed to secure the endurance of critical forest units (Aitken and Bemmels 2016). It is a must to integrate genetic data into conservation planning and management (Chen et al. 2022; Gaitán‐Espitia and Hobday 2021; Kardos et al. 2021). Being able to identify genetic provenances defined by climate and then obtaining seeds from other geographical areas may improve forest management strategies by increasing genomic and phenotypic diversity (Supple et al. 2018).

## 2 Material and Methods

All analyses that rested on R packages were run in R, version 4.4.3 (R Core Team 2025).

### 2.1 Populations Sampled

**FIGURE 1:**
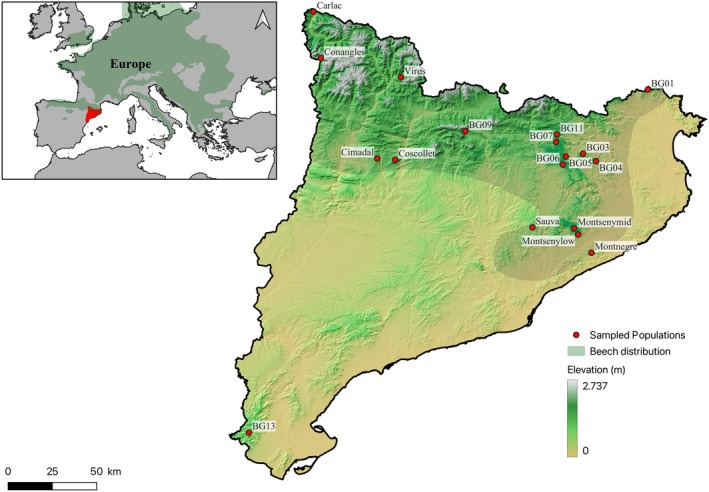
Potential distribution of *Fagus sylvatica* in Catalonia and Europe, and the sampling points.

**FIGURE 2:**
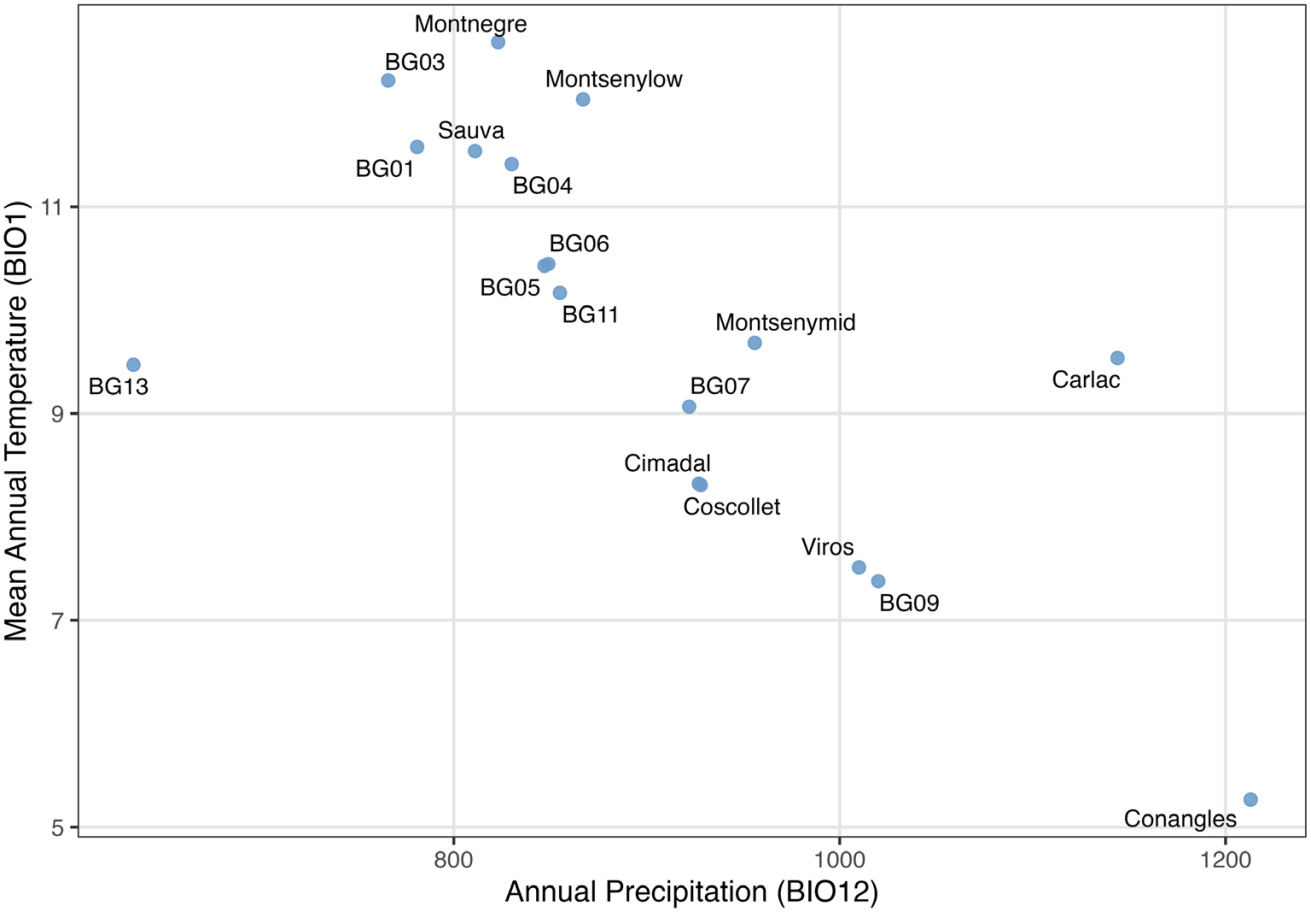
Climate niche of the studied *Fagus sylvatica* populations in Catalonia based on mean annual temperature (BIO1) and annual precipitation (BIO12). Climatic data extracted from WorldClim for the 1970–2000 period.

*F. sylvatica* covers 28,726 ha across a fragmented distribution in the study region (Figure 1). In many European countries, beech still represents the potential natural vegetation (PNV) (Hickler et al. 2012). While the highest abundance of this species is found in mid elevations (Jump, Hunt, and Peñuelas 2006), beech stands span from southern Scandinavia in the north to Sicily (central Mediterranean basin) in the south and from the Iberian Peninsula in the west to northwest Anatolia in the east (Houston Durrant et al. 2016), limited in the southern latitudes by water availability (Mátyás et al. 2010). In Catalonia, *F. sylvatica* found the limit for its ecological niche (Oliet et al. 2012), while in Europe it is a highly important broad‐leaved tree species, ecologically and economically (Lazic et al. 2024). Many of the populations sampled in this study are found on mountain ranges with complex topography that provide an adequate level of fog and air humidity that protects the species from the influence of the Mediterranean climate or at high elevations, providing sufficient rainfall to these forests (Figure 2).

### 2.2 DNA Sequence

The raw data used in this study consist of sequences obtained from pool sequencing (Schlötterer et al. 2014) of DNA from leaf material of 25 trees per population of *F. sylvatica* done by the Centre National de Séquençage (Evry, France). These leaves were sampled in 2017 from a total of 23 beech populations from Catalonia. After pooling all DNAs in an equimolar way, whole‐genome re‐sequencing was carried out using the Illumina GAIIx technology with a target 25× coverage; following sequencing, we trimmed and quality‐filtered raw reads, and then mapped them against the beech reference genome (Mishra et al. 2022) using the scripts reported in the BeechMarkET bundle (https://github.com/ulaszewski/beechMarkET). Quality of sequencing reads after trimming and quality filtering was inspected using FastQC (Andrews 2010). Following the production of BAM files and of one MPILEUP file (using SAMTOOLS v.1.13 (Li et al. 2009) with quality parameter ‐q 20), a .sync file was obtained using poPoolation2 (Kofler et al. 2011). The .sync file was imported as a pooldata object with the *popsync2pooldata()* function of Poolfstat R package (Gautier et al. 2022), using the following parameters: min.rc = 7, min.cov.per.pool = −1, max.cov.per.pool = 1e+06, noindel = TRUE. No coverage filtering was done initially to avoid removing a high number of SNPs (e.g., if a SNP had a high coverage for all the populations except one, it would be removed anyway if it fell outside the threshold). Then, the pooldata object was examined in R and filtered with the function *pooldata.subset()*, removing the population BG08 as its poolsize was extremely low (4).

Coverage filtering was done then on the resulting pooldata object, treating as missing data all values with a read coverage below 3 or above the haploid poolsizes (double the number of individuals). SNPs with missing data only, as well as populations with equal to or more than 70% missing data, were then removed. Then, the reference allele frequencies were obtained, and SNPs with more than one population with missing data were removed, imputing the remaining missing data with the median frequency per SNP, using a custom R script. Loci that had become monomorphic after the above treatments were removed to avoid errors in the GEAs methods. We also filtered a copy of the allele frequency matrix by removing all SNPs with missing data to be used in the Isolation‐by‐distance analyses.

### 2.3 Bioclimatic Variables

To obtain climatic data, we used the 19 bioclimatic variables from the WorldClim database (v2.1) for the years 1970–2000, with a resolution of 30 arcseconds (Fick and Hijmans 2017). We extracted the values for each population using the R package ‘raster’ (Hijmans 2010) with the *extract()* function. The climatic projections used in the Gradient Forest model correspond to the period 2061–2080 under all Shared Socioeconomic Pathways (SSP126, SSP245, SSP370 and SSP585) and for all periods (2020–2100) using SSP585, based on the General Circulation Model (GCM) provided by Max Planck Institute Earth System Model 1.2 for the ‘highres‐future’ (MPI‐ESM1‐2‐HR).

Prior to further analyses, we performed a correlation analysis to group the current‐time bioclimatic variables for the 18 populations, following the workflow described by Chang et al. (2022). To identify clusters of collinear predictors, we utilised the customised index suggested by Chang and collaborators based on the sum of squares (SS), considering SS as ‘the amount of information’. The index is calculated as:

(1) $$\frac{\Sigma {\mathrm{SS}}_{\mathrm{PC}1,n}}{{\mathrm{SS}}_{\text{Total}}-\Sigma {\mathrm{SS}}_{\mathrm{PC}1,n}}$$

In the equation, SS_PC1,*n*_ represents the sum of squares of the first principal component for each group of standardised variables, and SS_Total_ is the sum of squares of all standardised variables. Grouping variables with high collinearity increases the index value, while grouping variables with low collinearity decreases it.

Pairwise *R* ^2^ values among all standardised variables were first computed. Here, *R* ^2^ (coefficient of determination) corresponds to the squared Pearson correlation coefficient between two variables and represents the proportion of variance shared between them, ranging from 0 (no linear association) to 1 (perfect linear association). These *R* ^2^ values were converted into a dissimilarity matrix (1 − *R* ^2^), so that highly correlated variables had small distances. Hierarchical clustering (complete linkage) was then applied to this matrix, and the dendrogram was cut at different 1 − *R* ^2^ thresholds to generate alternative grouping configurations (e.g., a threshold of 0.2 may correspond to grouping variables with *R* ^2^ ≥ 0.8). For each configuration, a Principal Component Analysis (PCA) was performed within each group and PC1 was extracted to compute the index. Groups containing a single variable were excluded from synthetic component construction. The PC1 of each retained group was used as a synthetic predictor in subsequent analyses.

To further refine the dataset, we applied variable selection based on the Variance Inflation Factor (VIF), removing synthetic predictors with VIF > 5 to reduce residual multicollinearity. VIF is a diagnostic measure in regression analysis that quantifies how much the variance of an estimated regression coefficient is inflated due to linear dependence among predictors. When predictors are uncorrelated, VIF = 1; increasing multicollinearity results in progressively larger VIF values. After filtering, we assessed the cumulative variance explained by the retained synthetic predictors to ensure that they adequately represented the structure of the original environmental dataset.

### 2.4 Isolation‐by‐Distance (IBD)

*F*_ST_ values were computed using the *compute.fstats()* function from the ‘Poolfstat’ package. Then, to test whether populations followed a pattern of genetic differentiation that covaried with geographical distance, we performed a Mantel's test using the R package ‘vegan’ (Oksanen et al. 2024), with one million permutations to assess the significance of the correlation.

### 2.5 Genotype–Environment Association (GEA) Analyses

Using allelic frequencies, we carried out various genotype–environment analyses to assess whether beech populations have potentially locally adapted to the Mediterranean‐to‐Alpine climate gradient of Catalonia, and if so, identify which loci of the genome are under selection.

The first part of the GEA consisted of running a Latent Factor Mixed Model (LFMM) (Frichot et al. 2013). An LFMM analysis is based on a statistical model that uses regressions to evaluate the association of the response variable (allele frequency) and a set of environmental variables. This model considers the population structure as the latent factor or factors. To estimate the adequate number of latent factors, we applied a Principal Component Analysis (PCA) to allele frequencies, then observed the number of PCs that can explain the population structure, which are determined with the ‘elbow’ method based on the scree plot of PCs eigenvalues (Caye and Francois 2018). PCA was computed with the ‘pcadapt’ R package (Luu et al. 2017; Privé et al. 2020). We used the ‘lfmm’ package (Caye and Francois 2018) from R via the *lfmm_ridge()* function, which computes regularised least squares estimates for latent factor mixed models using a ridge penalty.

The *p*‐values obtained from LFMM were converted into *q*‐values with the R ‘qvalue’ package (Storey and Bass 2023). We used a False Discovery Rate (FDR) (Storey 2010) of 0.05 to identify significant correlations.

A Redundancy Analysis (RDA) was also conducted to detect loci potentially under selection. This multivariate ordination technique is known for its low false‐positive rate and high detection power in identifying genetic variation with adaptation signatures (Capblancq and Forester 2021). RDA assesses how groups of loci covary with multivariate environmental gradients and is particularly advantageous for accounting for population structure, which can confound signals of selection. To perform all RDA analyses, we followed the instructions provided in https://github.com/Capblancq/RDA‐landscape‐genomics.

In this study, we performed a partial RDA using the first two principal components (PC1 and PC2) from a PCA of allelic frequencies as covariates to capture population structure effects. Additionally, a simple RDA was conducted with SNPs as response variables and synthetic bioclimatic variables as explanatory variables to directly assess the relationship between genetic variation and environmental predictors.

To identify candidate loci, we applied the statistical framework described by Capblancq et al. (2018). RDA axes were selected based on their proportion of explained genetic variation. Then they were used to calculate Mahalanobis distances for each SNP. These distances were scaled using a robust covariance matrix estimator to account for potential significant SNPs. The inflation factor was calculated to correct for overdispersion in the Mahalanobis distances, enabling a Chi‐squared test for statistical significance. *p*‐values for each SNP were computed and subsequently adjusted for FDR control using the *q*‐value distribution.

SNPs with a *q*‐value below 0.05 were considered candidates under selection. The computations were implemented using the custom R function *rdadapt()* provided in the pipeline, which automates the statistical testing procedure, including Mahalanobis distance calculation, inflation factor correction, Chi‐squared testing and *q*‐value estimation.

We also used BayPass 2.41 CORE model (Gautier 2015) to obtain locus‐specific population differentiation and detect which outlier SNPs matched the GEA‐significant SNPs. This consisted of running the core model with the filtered set of SNPs. We detected outlier SNPs showing extreme divergence between genetic clusters using the XtX statistic, which is a Bayesian analogue of *F*_ST_ and explicitly models population covariance based on a Mahalanobis distance. The underlying models in this software explicitly account for (and may estimate) the covariance structure among the populations' allele frequencies that originates from the shared history of the populations under study. We used the following parameters: ‐npilot 25 ‐pilotlength 1000 ‐burnin 100,000 ‐nval 2500 ‐thin 40. The parameter setting followed the configuration proposed by Leblois et al. (2018). Pseudo‐observed data (POD) was simulated using the function *simulate.baypass()* to construct null distributions of the XtX values, thus obtaining a threshold value for the analysis, determined by the 99.5 quantile of POD XtX.

### 2.6 Gene‐Ontology (GO) Term Enrichment Analysis

We conducted a GO term enrichment analysis to identify functions for genes that are specifically targeted by selection. To do this, we used the GO annotations of coding sequences (CDS) from the annotated genome Bhaga (Mishra et al. 2022). We first computed the counts of each GO term (e.g., protein phosphorylation, nucleic acid binding, membrane) in each of the GO levels (a. biological process; b. molecular function; c. cellular component) reported for all annotated CDS; next, we identified the CDS that contain the significant SNPs obtained with the GEAs analyses. To test for enrichment of a specific GO term, we used the GO term counts in the whole genome as the ‘expected’ distribution, and the counts of each GO term (vs. all others) in the significant SNPs subset as the ‘observed’ distribution and applied a binomial test. This allowed us to test whether there was a significant deviation in the frequencies of GO terms we obtained and determine if there has been a selection for a specific type of gene.

### 2.7 Gradient Forest (GF), Allelic Turnover and Genetic Offset

Methods such as LFMM, RDA or BayPass AUX model are designed to test linear associations between environmental variables and allele frequencies. To observe non‐linear relationships and allele frequency turnover patterns that occur at a given point of the environmental gradient, we applied a Gradient Forest (Pitcher et al. 2011). Gradient Forest consists of a non‐parametric machine‐learning model that uses regression trees to establish patterns in the turnover of the genetic composition, using non‐linear functions to determine the change of frequencies along an environmental gradient (Fitzpatrick and Keller 2015).

We computed the model with a set of reduced (non‐synthetic) variables, eliminating the ones that had a high Pearson's correlation coefficient (|*r*| > 0.8), as suggested in Vanhove et al. 2021, with the function *findCorrelation()* from the R package ‘caret’ (Kuhn 2007) (Figure S1). Thirteen variables were eliminated, leaving us with BIO2 (Mean Diurnal Range), BIO4 (Temperature Seasonality), BIO5 (Max temperature of Warmest Month), BIO8 (Mean Temperature of Wettest Quarter), BIO9 (Mean Temperature of Driest Quarter) and BIO18 (Precipitation of Warmest Quarter). Apart from the climatic variables, in the GF model, we used Principal Coordinates of Neighbour Matrices (PCNMs) to account for the spatial effect. These variables were obtained with the function *pcnm()* from the ‘vegan’ package (Oksanen et al. 2024) in R. As suggested in Gugger et al. (2021), we used only half of the PCNMs that showed positive eigenvalues, avoiding overparameterizing the model with too many unnecessary variables.

Then the transformed environmental variables were reduced into multivariate synthetic variables using a PCA, allowing us to visualise the GF results in a map assigning a Red–Green–Blue (RGB) scale of colour palette to the first three components of the PCA, as suggested in Vanhove et al. (2021). A map with the expected allele turnover corresponding to climatic conditions defined in each grid cell of the raster is then generated for the *F. sylvatica* distribution in Catalonia (adapted from the European distribution provided by https://www.euforgen.org/species/fagus‐sylvatica/). To classify the genetic composition into clusters we used a K‐means analysis utilising the RGB values and selecting the cluster with the lowest silhouette.

Using the Gradient Forest model, we estimated the *genetic offset* (Ellis et al. 2012) for the regional beech distribution using all SSPs available for the 2061–2080 period and for SSP585 for all periods, ranging from 2020 to 2100. This is obtained by calculating the Euclidean distances between the current allele frequencies and the required ones in the future based on the climate predictions and the associations determined by Gradient Forest. Genetic offset provides a way to predict how far from the optimum the genetic composition of the studied populations will be in the future, assuming that the gene–environment correlation found nowadays is optimal. To check for differences between offsets of different SSPs, we used a Conover–Friedman test (Conover 1999).

However, using only this index can result in incorrect conclusions when comparing it between different forests. The offset informs about the distance between current and future gene–environment optimums, but it does not consider that Gradient Forest is a non‐linear model. Thus, the required genetic change may not be always equal along the timeline in which we do the prediction—if we predict the offset for 2080, this value might be reached in several different ways (e.g., rapid change during the first half and slower change at the last half). The order of events in the timeline is important, especially for forest management units to predict when interventions are needed and how much time they have left until the situation worsens. To achieve this, we should not only correlate the genetic offset with the environmental change but also relate it to time. Here, we developed a sophisticated yet not overly complex approach of doing so:

Genomic offset can be written as Δ*G*_*G*1→*G*2_, where *G*1 and *G*2 are the starting and final values of genomic state; analogously, we can define Δ*E*_*E*1→*E*2_ for the corresponding environmental change.

We first define a ‘rate’ of genomic offset as

(2) $$r_{G}=\frac{{\mathrm{\Delta G}}_{G1\to G2}}{{\mathrm{\Delta E}}_{E1\to E2}}$$

Moving to infinitesimals, this becomes, for arbitrary small values of *γ* and *ε*,
(3)

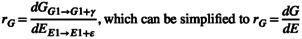




Because *E* varies in time according to a function determined by climate change scenarios, one can also define an empirical function describing the velocity of environmental change, for an arbitrarily small value of *τ*:

(4) $$v_{E}=\frac{{\mathrm{dE}}_{E1\to E1+\varepsilon }}{{\mathrm{dt}}_{t1\to t1+\tau }},\text{which}\;\mathrm{can}\;\mathrm{be}\;\text{simplified to}\;v_{E}=\frac{\mathrm{dE}}{\mathrm{dt}}$$

Finally, one can compute an empirical function for the ‘required evolutionary rate’ (i.e., the evolutionary rate that is instantaneously required to cope with environmental change) as:

(5) $$\text{REvoRate}=\frac{\mathrm{dG}}{\mathrm{dt}}=\frac{\mathrm{dG}}{\mathrm{dE}}\cdot \frac{\mathrm{dE}}{\mathrm{dt}}$$

This ‘instantaneous’ evolutionary rate is therefore computed based on the modelled, empirical relationships between genetic and environmental change, on the one hand, and between environmental change and time, on the other hand. This quantity describes genomic offset per unit time and complements the information conveyed by the commonly used relationship between genetic composition and environment, which is the foundation of genomic offset. To compute REvoRate, one would first choose the size of the time step, compute each component of the product given in Equation (5) for each time step, and multiply them. The resulting curve will represent REvoRate as a function of time.

In our case, we computed the baseline REvoRate, which is the minimum rate for a population to transition from one period to the next. To do this, we computed the increment between the genetic offset of the period at ‘*t*’ and the offset at ‘*t* + 1’. This way, we obtain the baseline required evolutionary rate, as any other value further from the genetic composition implied by the offset would require a higher rate, as it would have to overcome a steeper path in the same amount of time. We computed the discrete version of this rate, as using bidecadal periods is more logical than using the instantaneous rate, which would be preferred if annual predictions were used. We used the year at each period midpoint to compute the differences between periods.

## 3 Results

### 3.1 Data Acquisition

After equimolar pooling, DNA concentrations varied between 3.2 and 5.3 ng/μL. DNA sequencing provided between 80 and 116 million reads, for an expected coverage varying between 13X and 41X. No sequence was overrepresented, and per‐base sequence quality was close to top (40) for all bases except the first 1–5 of each which may have quality scores as low as 32 but not lower.

Prior to any filtering of the pooldata object we obtained a mean coverage of 3.08 for the conglomerate of 23 populations. The maximum mean coverage was 7 (BG01), and the minimum mean value of coverage was 0.52 (BG02). Initially, we obtained 5,894,627 variants, and the mean poolsize for the 23 populations was equal to 20.43 trees.

Firstly, we excluded BG08 due to a very low pool sample size (four individuals). Then, after analysing the levels of missing data of every population in the coverage matrix, Boavi, Montsenyhigh, BG02 and BG12 were removed because they surpassed the 70% missing data threshold.

With the remaining 18 populations, we removed all SNPs with more than 1 population with missing data, imputed the remaining missing data, and removed the monomorphic loci. From the initial 5,894,627 SNPs, we kept a total of 356,223 SNPs.

### 3.2 Bioclimatic Variables

A clustering analysis of the 19 environmental variables for the 18 populations showed 6 groups: BIO12 + BIO13 + BIO16 + BIO19; BIO14 + BIO15 + BIO17 + BIO18; BIO5 + BIO8; BIO1 + BIO4 + BIO6 + BIO10 + BIO11; BIO9; BIO3 + BIO2 + BIO7. Then a PCA was done for the 5 groups with more than one variable to obtain PC1 to be used as a synthetic variable, and BIO9 was used alone. Pairwise correlations between the PC1s (and BIO9 values) were calculated, obtaining a maximum absolute correlation of *r* = 0.75. Then, VIFs were calculated for each set of variables, dropping the groups BIO1 + BIO4 + BIO6 + BIO10 + BIO11 and BIO12 + BIO13 + BIO16 + BIO19 as they resulted in VIF > 5. The percentage of explained variance for the three remaining groups of variables was then checked, observing that synthetic variables described around 90% of the variance in each group (Table S1). With the final groups of variables, we obtained low‐intercorrelated groups with the highest absolute correlation being at *r* = 0.61 between BIO5 + BIO8 versus BIO14 + BIO15 + BIO17 + BIO18 (performing analyses using PC axes from a PCA based on bioclimatic variables returns similar results; not shown).

### 3.3 Isolation‐By‐Distance

**FIGURE 3:**
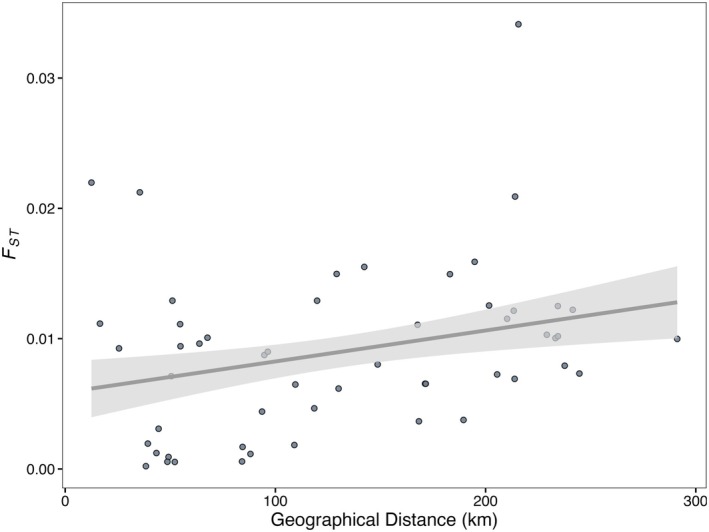
Mantel test results obtained with the correlation of geographic distance with genetic differentiation (*F*_ST_) (Mantel's *r* = 0.4173, *p*‐value = 0.047). Negative *F*_ST_'s were converted to 0 to perform the Mantel test but were not plotted in the linear regression. While we converted negative *F*_ST_ values–which can occur in Pool‐seq data–to 0 to perform the Mantel test, we considered not to add converted values in the plot to avoid misleading visual interpretations.

We obtained 170,774 SNPs without missing data by filtering the pooldata object and not applying imputation. Then, we computed *F*_ST_'s and performed a Mantel test that resulted in a positive trend in the correlation (Mantel's *r* = 0.4173, *p*‐value = 0.047; Figure 3) between geographical and genetic distance, even though that *p*‐values varied around 0.05 depending on the permutation.

### 3.4 Genetic‐Environment Association (GEA) Analyses

**FIGURE 4:**
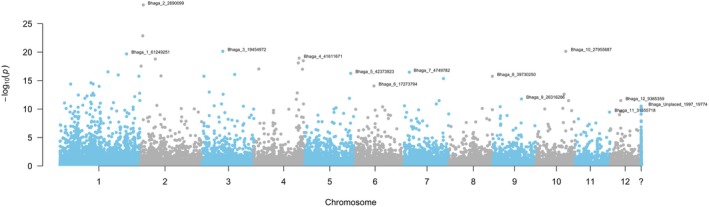
Manhattan plot of the *q*‐values obtained by LFMM with the BIO2 + BIO3 + BIO7 synthetic variable. Name of the lowest *q*‐value SNP is displayed per chromosome. The last unit ‘?’ groups all unplaced SNPs.

A PCA was conducted on the allele frequencies to identify latent factors within the populations and to assess the potential influence of population structure. The PCA revealed two latent factors (*K* = 2; Figure S2). Subsequently, LFMM was applied with two latent factors, incorporating three synthetic variables alongside BIO9. The *p*‐values were converted into *q*‐values, and a total of 9799 SNPs were identified as significantly associated with the environment at a false discovery rate (FDR) of 0.05 (Table 1). Notably, BIO2 + BIO3 + BIO7 exhibited the highest number of significant associations, with 4642 significant SNPs. These associations were widely dispersed across the genome (Figure 4), highlighting the broad genetic response to environmental variables. Some SNPs appeared to be associated with more than one variable. Twenty‐seven SNPs resulted in being significant across all environmental predictors.

**TABLE 1:** Number of SNPs associated with each variable and the total of unique SNPs associated with the environmental variables.

BIO14 + BIO15 + BIO17 + BIO18	4523
BIO2 + BIO3 + BIO7	4642
BIO5 + BIO8	779
BIO9	2471
Unique SNPs	9799

With RDA, we also identified thousands of significant SNPs associated with the environment. Partial and simple RDA detected 13,558 and 17,207 significant SNPs (FDR = 0.05), respectively; in both cases, SNPs were scattered across the genome as in the LFMM. *Rdadapt()* function was run using the first 2 axes of the RDA in both cases (Figures S3 and S4).

### 3.5 BayPass

This method identified 109 SNPs as putative outliers based on their XtX values, a measure of genetic differentiation that accounts for population structure covariance. To determine the significance threshold, we employed a POD approach, generating a null distribution of XtX values under neutrality. SNPs exceeding this empirical threshold were classified as outliers, suggesting they may be subject to selection rather than selectively neutral factors, such as genetic drift. Among them, 54 SNPs were uniquely detected by BayPass, highlighting loci exhibiting strong differentiation among populations but not necessarily linked to the environmental variables we used. Conversely, 22 SNPs were consistently identified across BayPass, LFMM and RDA‐based approaches, indicating limited but notorious overlap between differentiation‐based and environmental association methods, and 5 SNPs were detected across all methods.

### 3.6 Gene Ontology Term Enrichment Analysis

Analysis of all the significant SNPs detected by at least one of the four linear GEA methods revealed significant enrichment for a diversity of gene functions (Table S2). The most enriched term was the Cellular Component ‘membrane’ followed by the Molecular Function categories ‘binding’ and ‘nucleic acid binding’. The most enriched Biological Processes terms included ‘primary metabolic process’, ‘DNA integration’, and several others related to metabolism. We also observed some depleted terms, including ‘chloroplast’, ‘oxidoreductase activity’ and ‘ATP binding’, among others.

### 3.7 Gradient Forest Analysis and Genetic Offset Predictions

The Gradient Forest model was run with the reduced set of bioclimatic variables to avoid high correlations between them instead of using the synthetic variables. The bioclimatic variables explain the model variance with a mean *Importance* = 10.82%.

**FIGURE 5:**
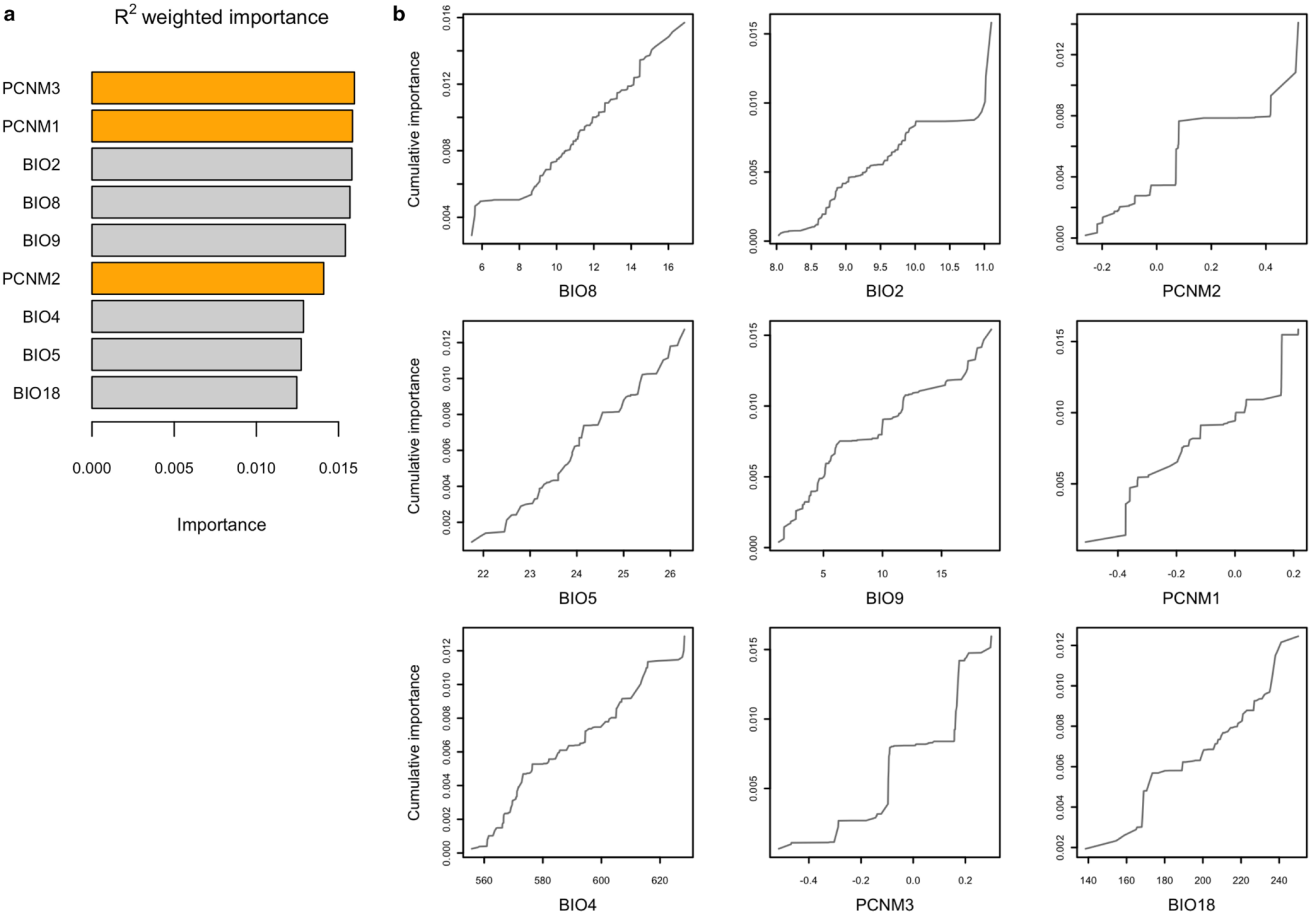
(a) *R* ^2^‐weighted importance of bioclimate (grey) and spatial variables (orange). (b) Cumulative importance of allelic turnover for the environmental gradient of every variable. Ordinates represent the cumulative sum of *R* ^2^‐weighted split importance across all SNP regression trees, reflecting aggregated allele‐frequency turnover along each bioclimatic variable.

We observe in Figure 5 that the cumulative change in allele frequencies does not correlate linearly with the environmental variables and that not all of them vary equally along the gradient. Principal Coordinates of Neighbour Matrices (PCNM3 and PCNM1) stand out as the two most important spatial variables, indicating that geography plays an important role in determining the genetic composition of the forests, followed closely by Mean Diurnal Range (BIO2), Mean Temperature of Wettest Quarter (BIO8), and Mean Temperature of Driest Quarter (BIO9). Precipitation of the Warmest Quarter (BIO18) is the least important variable, but it has a similar *R* ^2^ value as Temperature Seasonality (BIO4) and Max Temperature of Warmest Month (BIO5), indicating that, while not as important as the other variables, these also play a role in the allelic turnover.

**FIGURE 6:**
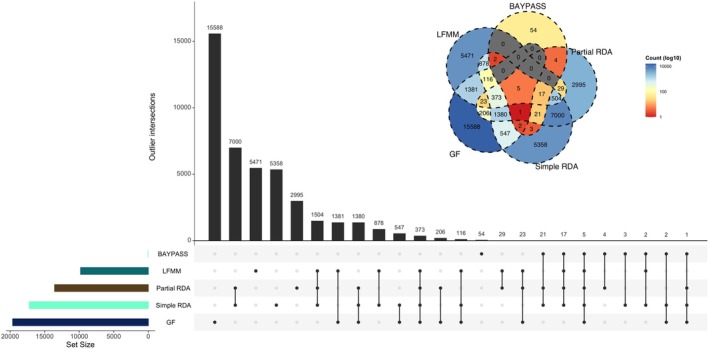
Upset plot and Venn diagram of all the significant SNPs detected by the five different methods. Note that all SNPs detected by Gradient Forest as *R* ^2^ ≥ 0.1 were considered to potentially be significantly associated with the environment, thereby excluding loci with low explanatory power.

Across all GEA and differentiation approaches, we observed both method‐specific and shared candidate SNPs (Figure 6). GF uniquely identified 15,588 SNPs. The largest shared intersection comprised 7000 SNPs jointly detected by simple and partial RDA. Extending this overlap, 1504 SNPs were shared by both RDAs and LFMM, a method that additionally shared 1381 SNPs with GF, while both RDAs jointly shared 1380 SNPs with GF. Across all five methods, five SNPs were consistently detected, and the four GEA approaches detected 373 common SNPs. A total of 42,958 SNPs were identified across all methods (Table S3).

**FIGURE 7:**
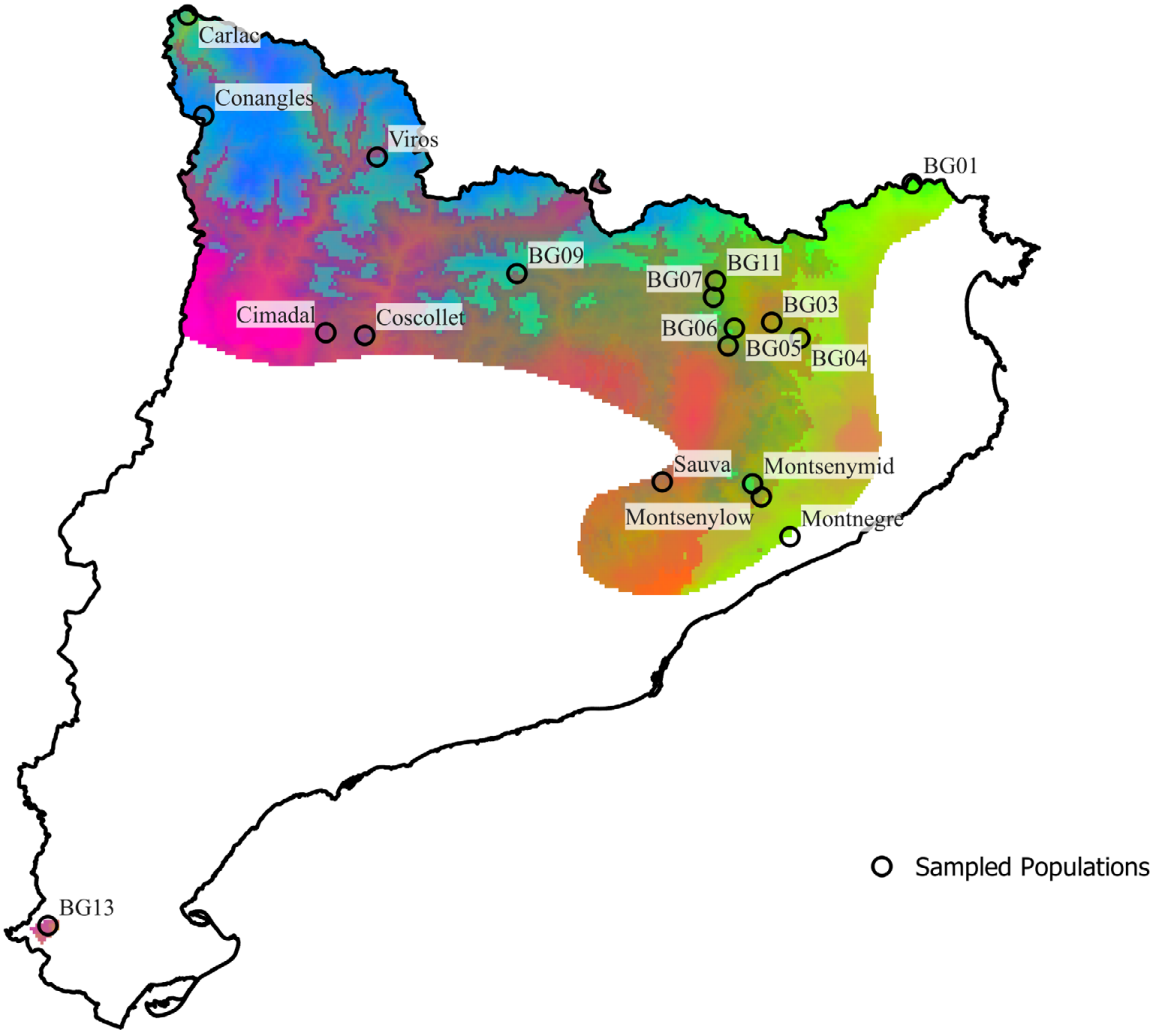
Predicted allelic turnover across the current landscape based on the Gradient Forest model. The first principal components of the GF‐transformed environmental space were mapped to an RGB colour scale (PC1 = red, PC2 = green, PC3 = blue). Colour combinations reflect multivariate turnover in predicted allelic composition; regions with similar colours are expected to have a similar predicted genetic composition. Notice that population BG13 is located at the southernmost tip of the map.

After identifying these correlations, using the GF model we were able to map the allelic turnover for significant SNPs over the potential distribution of beech in Catalonia (Figure 7). Contrasting patches of pixels with similar predicted genetic composition are apparent. Under the RGB colour scale, pixels with more similar predicted genetic composition display more similar colours, enabling visual assessment of spatial variation and shifts in genetic composition. We observed a clear difference between the northwest and southeast regions in the predicted genetic composition, as well as suture zones between areas of divergent genetic composition. We did a K‐means based on the map's RGB values, dividing the region into three clusters that correspond to three climate‐predicted genetic groups (see *k*‐means map at Figure S5).

**FIGURE 8:**
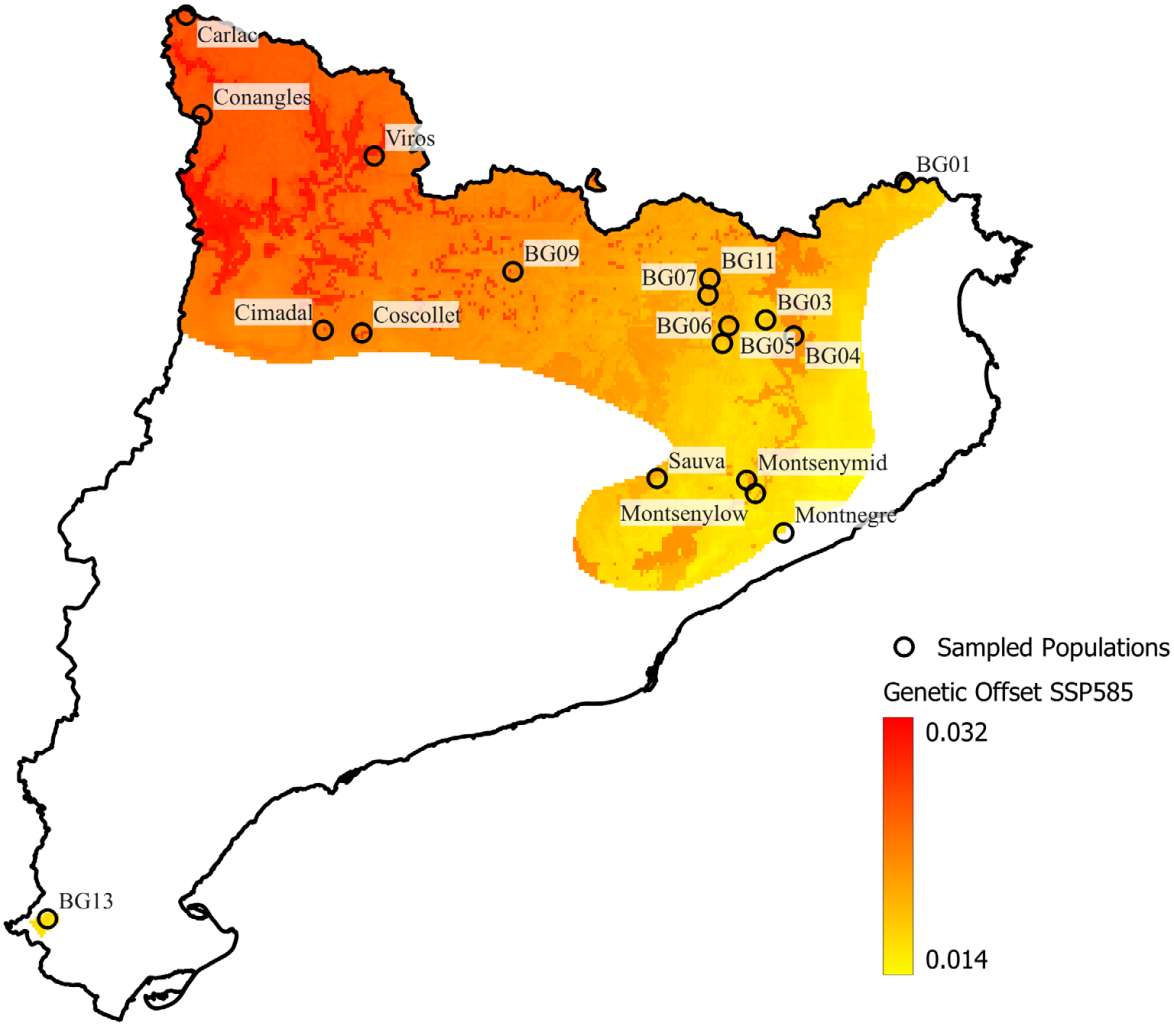
Genetic offset for SSP585 was mapped for the distribution of beech in Catalonia; the northwest region shows a worse scenario than the southeast area. Carlac (the most northern population and located in the Atlantic basin) shows a lower offset than other proximal populations.

We mapped the predicted genetic offset across Catalonia for all the scenarios available from the years 2061–2080 (Figure 8; Figure S6). This measure corresponds to the Euclidean distance between the current genetic composition (under the postulate that it is optimal under the current climate), and the expected composition under the future climate. At SSP585 we observe a clear trend that shows higher offsets at locations that are at the same time more western, more northern, farther from the coast, and surrounded by higher mountains, as climate shifts are expected to be bigger there than near the coast. Even a significant correlation between offset and latitude was detected (*p*‐value = 0.011; Figure S7). Regarding the different climate scenarios, SSP126 shows the lowest offset values, incrementing over each climate scenario and with slight but significant differences between all scenarios as stated by the Friedman‐Conover test (Table S4).

Although genetic offset provides a useful measure of the magnitude of environmental displacement, it does not account for the temporal dynamics of climate change. Because adaptation must occur within a finite time window, we further calculated the Required Evolutionary Rate (REvoRate)—the amount of genetic change per year needed to keep pace with projected environmental shifts. Integrating this metric allowed us to identify populations at risk of temporal maladaptation.

**FIGURE 9:**
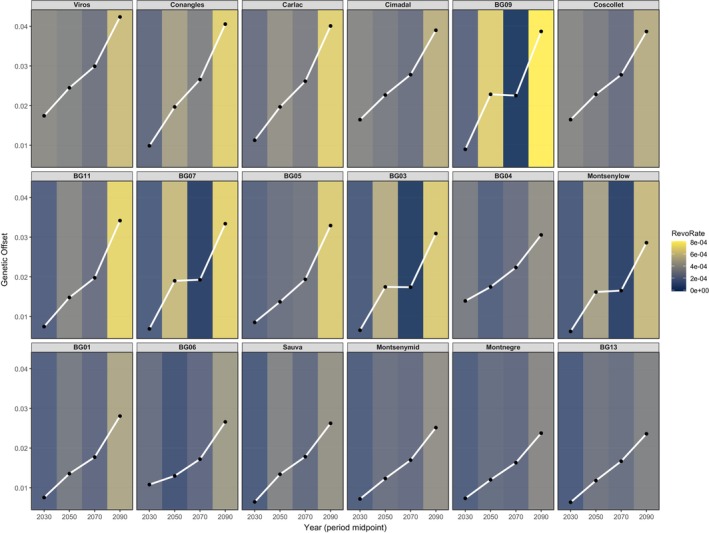
Genetic offset mapped across time and the REvoRate for each period (computed from period to period).

Joint examination of offset and REvoRate trajectories across time (Figure 9) revealed that while Pyrenean and pre‐Pyrenean populations consistently show the highest offsets for the last period, this does not always correlate with also requiring the highest rates from period to period. Period one is completely defined by the fact that the offset regresses linearly with the REvoRate, as it does not depend on the last period offset (which would be considered zero) so the relationship between the two indexes remains linear. However, it is in the second period that we start to see differences when ordering the population with the highest offset against populations with the highest required rates to transition from period one to two. Viros is the population with the highest absolute offset in every period; however, from period two onwards it is never the forest with the highest required evolutionary rate. The highest REvoRates are shared between BG09 (periods two and four) and Conangles (period three). Viros, however, has much lower REvoRate levels during the second and fourth period—corresponding to the eighth highest rate—and upper end values for the third period (fourth highest rate).

Notably, however, the temporal perspective uncovered additional patterns invisible in static offset maps: the Mediterranean‐basin populations (Cimadal, Coscollet and Viros) exhibited early peaks in required rates during 2021–2040, indicating rapid near‐term adaptation demands. In other cases, such as BG09, BG07, BG03 and Montsenylow, the offset remains stationary during the transition from the 2nd to the 3rd period, rising again later and provoking high rates on the passage to the fourth period (Figure 9).

## 4 Discussion

Our results point out that climate change has become a serious issue threatening the habitats available for *F. sylvatica* , making no exception for this species from the rest of the global biodiversity (Coristine and Kerr 2011). In the next decades, the maladaptation registered in this study could decrease the fitness of beech populations in the species' driest range edge, increasing the risk of contributing to the growing number of local extinctions in many plants and animals in the mid‐term future (Holzmann et al. 2023). The small geographical scale at which we have analysed genomic offset allowed us to identify variations of threat level depending on local climatic conditions and genetic composition.

Genetic data and evolutionary‐based modelling of future responses of populations to climate change can usefully complement forestry inventories and projected changes in forest composition. Indeed, understanding the genetic structure of the populations provides a more realistic forecast of geographical range shifts by considering genetic variability (Gotelli and Stanton‐Geddes 2015). Such results are a major contribution to the improvement of conservation and management actions. To carry out our analyses, we have used statistical models already tested and used previously for similar purposes (e.g., Chu et al. 2021; Frichot et al. 2013; Gugger et al. 2021; Jia et al. 2020).

We have uncovered many significant linear and non‐linear associations between current climate conditions and allele frequencies, suggesting that beech forests in Catalonia have potentially undergone local adaptation to the past and present environment.

### 4.1 IBD Shows a Weak but Positive Trend

We obtained a weak but significant correlation between genetic and geographic distances, as described in similar cases: Krajmerová et al. (2017), Müller et al. (2018) and Pluess et al. (2016). While we could not identify the local structure for each population due to the use of pooled data, we determined *K* = 2 latent factors for the ensemble of populations, indicating a weak structure and small genetic differentiation between all the populations studied (*F*_ST_ = 0.0031). This shallow spatial structure value could be caused by low genetic isolation between the populations (Gutiérrez et al. 2005), driven by long‐distance gene flow (coincident with the IBD results) or other factors that remain unstudied for these populations (e.g., historical bottlenecks or founder effects).

### 4.2 Characterisation of the GEAs

Comparing multiple analytical frameworks enables the identification of high‐confidence candidate loci, as each method relies on distinct statistical assumptions and model structures; reliance on a single approach may therefore introduce method‐specific biases. GEA methods are known to exhibit elevated false‐positive rates, even after stringent correction for multiple testing (Lazic et al. 2024). Consequently, concordance among independent approaches provides a pragmatic strategy to increase confidence in candidate SNPs and to mitigate the risk of spurious associations.

On the basis of a matrix factorization approach, LFMMs provide a unified framework for estimating the effects of environmental and demographic factors of genetic variation (Frichot et al. 2013). LFMMs differ from many other statistical tests proposed for detecting genes evolving under selection and local adaptation because they consider latent factors, which are unobserved variables. While many popular statistical tests to analyse population genetic data are based on a null model, which is completely non‐spatial (Meirmans 2012). LFMMs capture the part of genetic variation that cannot be explained by the set of measured environmental variables. Latent factors could result from the demographic history of the species, other environmental variables that are not used in the model or from IBD patterns (Frichot et al. 2013).

On the other hand, RDAs are based on multivariate regression and linear combinations of the environmental predictors that explain linear combinations of the SNPs (Capblancq and Forester 2021). We used both flavours of this method: a simple RDA fitted with the response and explanatory variable and a partial RDA that also adds covariates. Then, we compared them to check for overlaps between the two. This method provides a flexible framework for this type of analysis and has been used to map adaptive variation in common beech across the French Alps (Capblancq et al. 2020).

To complement the linear GEA methods, we used the BayPass CORE model, which is based on a Bayesian framework. This algorithm accounts for the covariance structure among populations by estimating a population covariance matrix, which helps correct for shared demographic history and genetic drift (Gautier 2015). This approach is particularly well‐suited for pool‐sequencing data and allows for the detection of outlier SNPs that exhibit unusually high differentiation, which may signal local adaptation. Using the pseudo‐observed data approach, which simulates data under the inferred neutral model and estimates an empirical distribution of XtX values, allowed us to set robust significance thresholds and identify candidate SNPs with greater confidence.

### 4.3 Polygenic Local Adaptation

Both linear GEAs point to a wide polygenic effect, detecting many loci dispersed across each chromosome. These results suggest potential local adaptation of *F. sylvatica* populations to the environmental gradient of Catalonia. This resonates with results from other areas of Europe (Meger et al. 2021; Modica et al. 2024; Lazic et al. 2024; Pfenninger et al. 2021; Postolache et al. 2021) that compared multiple habitats; indeed, European beech is found in many different environments and is therefore a good candidate for showing multiple kinds of adaptation to gradients (Tsiripidis et al. 2024).

Overall, we see that the ensemble of GEAs point to a polygenic structure, detecting thousands of SNPs putatively associated with the environment across the genome, with chromosome 1 being the one with the most signals.

### 4.4 GO Analysis

We performed a Gene‐Ontology Enrichment analysis with a binomial test, which revealed several terms (across the three Gene Ontology levels) as being over‐represented in the significant SNPs group. A variety of functional classes were found among the over‐represented groups, without a clear pattern and without any obviously meaningful link to the environmental gradients examined here.

### 4.5 The Drought‐Temperature Gradient Determines Allelic Turnover

In the LFMM we observed that temperature range, closely followed by the precipitation in the driest or warmest months of the year, is the main driver of allele frequencies of associated SNPs, thus determining the genomic composition of these populations. We observed a similar pattern in the Gradient Forest model, where the most important bioclimatic variable was the mean diurnal range, supporting the results of the linear model. Following closely were the mean temperatures of the wettest (BIO8) and driest (BIO9) quarters. While the last does not correlate highly with any other variable, BIO8 shows Pearson's *r* around 0.5 and 0.6 with precipitation seasonality and precipitation of the driest quarter, respectively, supporting once again the precedent of the LFMM.

### 4.6 Three Climatic‐Based Groups

The Gradient Forest model also allowed us to interpolate the allelic turnover and the genetic offset found in the populations studied to the rest of the distribution of the region. While the allelic turnover map shows us the difference in the expected genetic composition in all the territory, it does not provide a way to identify nor classify groups directly from the map. However, as each colour represents an exact genetic composition, and closer colours characterise similar genetic compositions, we did a K‐means analysis to identify which was the best number of clusters based on the lowest value of silhouette. It was not surprising to see the distribution divided into three main genetic provenances based on climate. We observed high levels of turnover in suture zones (e.g., valley slopes), where in a small geographical distance we projected many allelic differences.

### 4.7 Different Scenarios, Similar Offsets

Although we identified significant differences in genetic offset between the various climate scenarios, we observed a general trend: the more severe the scenario, the more similar the predicted genetic offsets become. It is only for SSP126 that offset values are much lower than the rest; the next possible pathways offer more similar offset, which, while different from one another, occupy the higher part of the range of values. This suggests that under harsher future conditions, distinct SSPs may lead to comparable biological consequences. Such a convergence resembles a *tipping point* pattern, where further deterioration yields diminishing differences in impact, indicating a potential threshold beyond which ecological or evolutionary responses become uniform.

### 4.8 Best Today, Worst Tomorrow—Greater Maladaptation in the Pyrenees

The climate currently experienced by populations found in the Pyrenees and other high‐altitude interior mountain ranges should match better the general beech requirements, relative to other Catalonian populations (Mazza et al. 2024), but they are expected to suffer the biggest shift in climate relative to the studied regions. While all populations are predicted to be maladapted to future climate conditions, genetic offset interpolation for the third period showed two major areas with mainly higher and lower offsets, distributed in the northwest and southeast, respectively. This indicates that the bigger shift in the north produced, as expected, higher values of genetic offset. Following this, we have identified a significant correlation between genetic offset and latitude. This leaves us with several stands far from their optimal genetic composition, in a region that nowadays is not at the limit of the drought threshold of the species. However, forests found closer to the Mediterranean Sea will not be as maladapted, primarily because warming and drying trends are projected to be weaker near the Mediterranean Sea rather than inland or the Pyrenees (Calbó et al. 2012).

To identify populations most exposed to rapid adaptive demands, we quantified genetic offset for each available period of the 21st century (2020–2041, 2041–2060, 2061–2080 and 2081–2100). From these, we derived the baseline REvoRate between consecutive periods. Examining both metrics jointly allowed us to pinpoint forests at higher risk of maladaptation—not only those facing large absolute offsets but also those requiring fast genomic shifts over short timescales. This distinction is critical: some populations (e.g., BG09; a population located in a pre‐Pyrenean mountain range with a similar climate niche to Viros) may not exhibit the highest offset values yet still demand rapid genetic change, which increases the likelihood of failing to reach the adaptive optimum in time. Thus, what matters is not only how far a population must shift genetically but also how fast it must do so.

Predicted offsets and REvoRates were consistently highest for Pyrenean and pre‐Pyrenean populations, with Viros (Pyrenean population) showing the largest offset across all periods. However, the ranking of populations differed between offsets and rates, indicating that a higher offset does not necessarily entail a higher required rate of change. For instance, Viros, Coscollet and Cimadal (located on the northwest study area in the pre‐Pyrenees and Pyrenees) displayed the highest offsets—and consequently the highest REvoRates—during the first period, when the initial offset was zero for all populations. Yet, in the following period, Viros retained the highest offset but dropped to eighth place in terms of REvoRate. Conversely, BG09 showed the second‐highest offset and the highest required rate, while BG07 (pre‐Pyrenees) ranked only seventh in the offset but still required a relatively fast evolutionary response.

During the transition from the second to the third period, several populations (BG09, BG07, BG03; an inland population, Montsenylow; a low altitude population in a pre‐littoral mountain range) appeared to enter a stationary phase, meaning that if they reach their predicted offset for the second period, they would remain close to the expected optimum under the third. Although this apparent stabilisation might seem advantageous, it may actually delay adaptation, forcing a sharp increase in REvoRate in the final transition (2081–2100). In contrast, Viros, despite its consistently high offsets, exhibits a steadier adaptive trajectory and avoids extreme rates of change later in the century.

Overall, these regions that currently provide suitable environments for beech are projected to become increasingly unfavourable by mid‐century, completing the results achieved with species distribution models (del Río et al. 2018). Importantly, not all populations follow the same maladaptation pathway; some experience large offsets gradually, while others face abrupt shifts that demand rapid evolutionary responses.

### 4.9 Looking Beyond End‐Point Mismatch

As exemplified above, assessments of maladaptation risk can change substantially when genetic offset is considered together with the required evolutionary rate, because populations are challenged not only by the magnitude of mismatch at the end of a period, but also by the trajectory and tempo of change leading to that endpoint. Although Viros and BG09 show similar absolute offsets for the second period (Viros = 0.0245; BG09 = 0.0225), the temporal distribution of mismatch differs between them. Under the baseline assumption that populations track the predicted optimum at each time slice, Viros would accumulate a larger share of its period‐2 displacement during the first transition, whereas BG09 would need to accomplish most of its period‐2 displacement within a single transition. Consequently, BG09 faces a higher REvoRate over the early interval—that is a more time‐compressed change—despite its slightly lower end‐point offset in period 2. The same happens for the last period, where BG09 shows the highest REvoRate of all, despite having the fifth highest offset of the 18 populations.

This contrast exposes a dilemma: determining the equilibrium between having to reach a large offset at a slower pace or having to achieve a smaller offset but at a faster pace. Which of the two, the total amount of change or the speed at which it needs to happen, will be more important is a crucial question to answer to complete the interpretation of these metrics. While genomic offset provides insight into the magnitude of change required, coupling it with REvoRate integrates the temporal component of adaptation, potentially leading to a comprehensive interpretation of population risk.

### 4.10 Caveats and Validation of Genetic Offset and REvoRate

Although genomic offset has become a widely used proxy for climate maladaptation, it is not a direct estimate of fitness decline. It quantifies a distance in predicted genomic space under the assumption that present‐day genotype–environment relationships reflect local adaptation and remain relevant under future climates (Francisco et al. 2025; Rellstab et al. 2021). Consequently, genetic offset is sensitive to model choice (e.g., linear vs. non‐linear response functions), the marker set used (candidate vs. genome‐wide), the treatment of confounding by demography and structure, and the environmental predictors and spatial resolution of the climate data—each of which can alter both absolute offset values and the ranking of populations.

A further limitation is extrapolation to no‐analogue climates. Methods such as GF may yield strong offsets when projected into environmental conditions outside the calibration range, where predictor interactions and non‐linearities are poorly constrained.

Because offset metrics ultimately rest on correlational GEAs, empirical validation is essential. In a recent study of *Taxus baccata* , genomic offset predictions were evaluated against multiple fitness‐related traits measured in a large common garden experiment, supporting a negative relationship between offset and performance; however, the authors also reported that genome‐wide SNP sets retained substantial predictive ability and that restricting analyses to climate‐associated loci did not markedly increase predictive performance, suggesting that offset‐based predictions may capture broader genomic structure in addition to putatively adaptive loci (Francisco et al. 2025). This result cautions against interpreting offset values as mechanistic measures of adaptive load without independent evidence, and illustrates why validation provenance trials, reciprocal transplants, or long‐term monitoring are critical whenever offset is used to guide management.

Our REvoRate metric inherits all the above sensitivities because it is a temporal derivative of genetic offset, and it can additionally amplify the noise by differentiating successive time slices. REvoRate should therefore be viewed as a relative indicator of the pace at which populations would need to track shifting optima, not as an estimate of selection intensity or realised adaptive capacity. Future developments could (i) propagate uncertainty through bootstrapping/ensembling of GEA models and climate projections, (ii) account for environmental novelty explicitly, and (iii) integrate demographic and gene‐flow constraints (e.g., effective size, migration) to better connect offset trajectories to the probability of evolutionary rescue (Ahrens et al. 2025; Rellstab et al. 2021).

## 5 Conclusions

We sought to determine if SNPs identified from the genome of *F. sylvatica* , a keystone tree species in Europe, show patterns that could point to local adaptation to the climate variation within Catalonia, the most southeastern region in the distribution of this species in Spain. Using a large set of SNPs and their reference allele frequencies, we identified several significant SNPs (integrating different GEA analyses) putatively linked to drought and temperature gradients. We also uncovered the current allelic turnover and quantified the expected genetic offset during different periods of the 21st century under SSP585. We introduced a temporal extension—the REvoRate—to capture the speed at which genomic change must occur to maintain climatic equilibrium.

This dual‐metric approach reveals that the magnitude of maladaptation risk (offset) and the temporal urgency of adaptation (rate) do not always coincide. Populations showing the highest offsets (e.g., Viros), may face lower short‐term rates of change, whereas other stands (e.g., BG09) must undergo faster genetic shifts despite more moderate offsets. Such patterns highlight that both the distance and the pace of the genomic change are crucial to assess adaptive capacity under rapid climate transitions.

The integration of REvoRate with genetic offset provides a more dynamic understanding of adaptive potential, identifying populations at greatest temporal risk and providing important information to advise management actions. Our results suggest that areas currently optimal for beech growth may become maladaptive by mid‐century, emphasising the urgency of incorporating genomic forecasts into conservation and reforestation strategies. Ultimately, coupling genomic offset with its temporal derivative provides a scalable framework to anticipate where and when evolutionary rescue may fail–insight essential for sustaining forest resilience in a rapidly changing climate.

## Funding

The BEECHGENOMES project (2017–2020) was funded by the France Génomique call and led by INRA‐URFM (Ivan Scotti). A.J. was supported by grants NE/V00929X/1, NE/S010041/1 and NE/G002118/1 from the Natural Environment Research Council. This study was supported by grants from the German Science Foundation (Th1632/18‐1) and the National Science Centre, Poland (2018/31/F/NZ2/01747). A.V.‐C. was supported by the European Union's Horizon 2020 Research and Innovation Programme under Marie Skłodowska‐Curie grant agreement no. 656300, and by the Ministry of Science, Innovation and Universities (Spain)—Ramón y Cajal fellowship (RYC2023‐045604‐I).

## Conflicts of Interest

The authors declare no conflicts of interest.

## Supporting information


**Figure S1:** Correlation chart of all the current‐time bioclimatic variables. The matrix summarises pairwise correlations among predictors and shows that several variables are strongly correlated, indicating substantial collinearity in the climatic dataset.
**Figure S2:** Scree plot of the PCA from the allele frequencies obtained with Pcadapt. The plot shows the variance explained by successive principal components and was used to assess how many axes summarise the major structure present in the genomic dataset.
**Figure S3:** Eigenvalues of the partial RDA. The distribution of canonical eigenvalues shows the amount of genomic variation explained by the constrained axes after accounting for the covariates included in the partial model, allowing evaluation of the relative importance of each retained axis.
**Figure S4:** Eigenvalues of the simple RDA. The eigenvalue distribution summarises the amount of variation explained by each canonical axis in the model without conditioning variables, providing a direct comparison of axis importance in the unconstrained analysis.
**Figure S5:** Map with K‐means preferred clusters based on the allelic turnover pattern. Each spatial unit is assigned to the cluster that best matches its predicted allelic composition, illustrating the main geographic structure of genomic turnover across the study area.
**Figure S6:** Genetic offset maps for 2061–2080 under all SSP scenarios. The panels show the spatial distribution of projected offset for each scenario, and the same colour scale is used throughout to allow direct comparison of offset magnitude and geographic pattern among scenarios.
**Figure S7:** Linear regression between genetic offset for 2061–2080 under SSP585 and latitude. The fitted model indicates a significant association between latitude and projected genetic offset (*p*‐value = 0.011; *R* ^2^ = 0.3438), suggesting a geographic trend in vulnerability under this climate scenario.
**Table S1:** Explained variance of the environmental synthetic variables and their loadings. The table reports the variance captured by each synthetic environmental variable together with the loadings of the original bioclimatic variables after sign rotation. These values identify the main climatic gradients represented by the reduced predictor set used in subsequent analyses.
**Table S2:** List of enriched/depleted GO terms. For each term, the table reports the ontology category, annotation frequency, and direction of enrichment or depletion in the analysed gene set. This summary highlights the functional categories that are overrepresented or underrepresented among the loci considered in the enrichment analysis.
**Table S3:** List with all significant SNPs, identified by their chromosome and position inside it, and by which method/s have they been detected. This table compiles the complete set of SNPs detected across the GEA approaches and Baypass used in the study, and allows comparison of concordance across methods.
**Table S4:** Friedman test followed by Conover post hoc pairwise comparisons among genetic offset values for the 2061–2080 scenarios. For each contrast, the table reports the *p*‐value and significance level, providing a formal comparison of differences in projected offset among climate‐change scenarios.

**Table S3:** eva70230‐sup‐0002‐TableS3_suppl

## Data Availability

Raw data are stored at the European Nucleotide Archive (ENA, https://www.ebi.ac.uk) under accession numbers PRJEB108434 (project) and ERR16699334 through ERR16699351 (run accessions of individual sequencing pools). The curated data (allele counts) as well as the code allowing to obtain them, are permanently stored at https://recherche.data.gouv.fr under https://doi.org/10.57745/IT8UOD. An R package developed to compute REvoRate based on the code we used can be found at https://github.com/JosepMorando/REvoRateR.
