# Supplementary material for: Re‐Interpreting Genetic Offset: Quantifying the Least Required Evolutionary Rate Under Climate Change at the Mediterranean Range Margin of European Beech
Source: Evol Appl. 2026 Apr 8;19(4):e70230. doi: 10.1111/eva.70230 (PMC13058577; doi:10.1111/eva.70230)
Supplement: Supplementary file 1 — Figure S1: Correlation chart of all the current‐time bioclimatic variables. The matrix summarises pairwise correlations among predictors and shows that several variables are strongly correlated, indicating substantial collinearity in the climatic dataset. Figure S2: Scree plot of the PCA from the allele frequencies obtained with Pcadapt. The plot shows the variance explained by successive principal components and was used to assess how many axes summarise the major structure present in the genomic dataset. Figure S3: Eigenvalues of the partial RDA. The distribution of canonical eigenvalues shows the amount of genomic variation explained by the constrained axes after accounting for the covariates included in the partial model, allowing evaluation of the relative importance of each retained axis. Figure S4: Eigenvalues of the simple RDA. The eigenvalue distribution summarises the amount of variation explained by each canonical axis in the model without conditioning variables, providing a direct comparison of axis importance in the unconstrained analysis. Figure S5: Map with K‐means preferred clusters based on the allelic turnover pattern. Each spatial unit is assigned to the cluster that best matches its predicted allelic composition, illustrating the main geographic structure of genomic turnover across the study area. Figure S6: Genetic offset maps for 2061–2080 under all SSP scenarios. The panels show the spatial distribution of projected offset for each scenario, and the same colour scale is used throughout to allow direct comparison of offset magnitude and geographic pattern among scenarios. Figure S7: Linear regression between genetic offset for 2061–2080 under SSP585 and latitude. The fitted model indicates a significant association between latitude and projected genetic offset (p‐value = 0.011; R 2 = 0.3438), suggesting a geographic trend in vulnerability under this climate scenario. Table S1: Explained variance of the environmental synthetic variables and thei [file EVA-19-e70230-s001.docx]

**SUPPLEMENTARY**

**Figure 1.** Correlation chart of all the current-time bioclimatic variables.


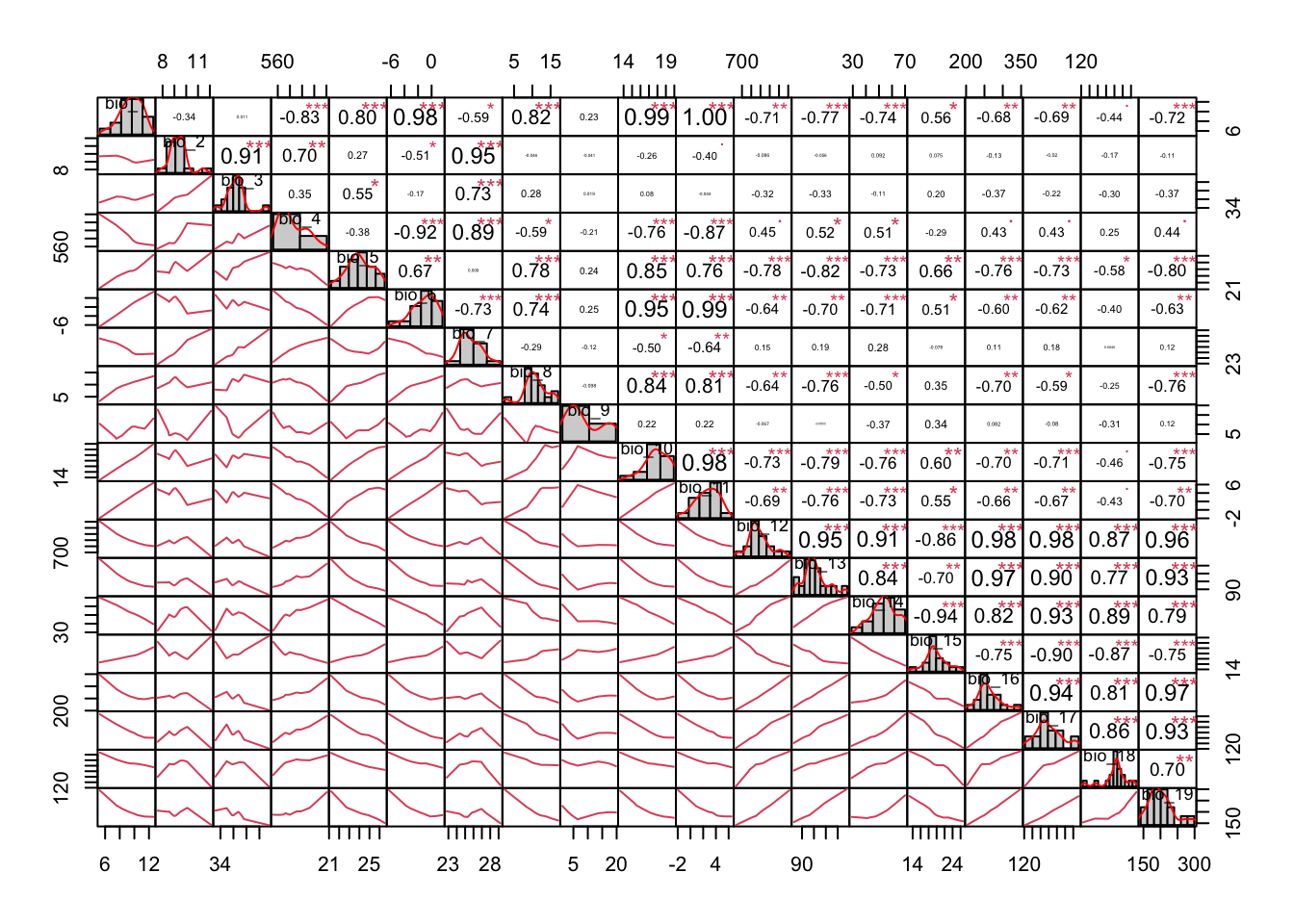


**Table 1.** Explained variance of the environmental synthetic variables and their loadings.

| *BIO14+BIO15+BIO17+BIO18* | 0.923 |
| --- | --- |
| *BIO2+BIO3+BIO7* | 0.910 |
| *BIO5+BIO8* | 0.889 |

Loadings Env PCs (after sign rotation)

| *BIO14* | *BIO15* | *BIO17* | *BIO18* | *BIO2* | *BIO3* | *BIO7* | *BIO5* | *BIO8* | *BIO9* |
| --- | --- | --- | --- | --- | --- | --- | --- | --- | --- |
| -0.507 | -0.502 | -0.499 | -0.489 | 0.604 | 0.559 | 0.567 | 0.707 | 0.707 | - |

**Figure 2.** Screeplot of the PCA from the allele frequencies obtained with Pcadapt.

**
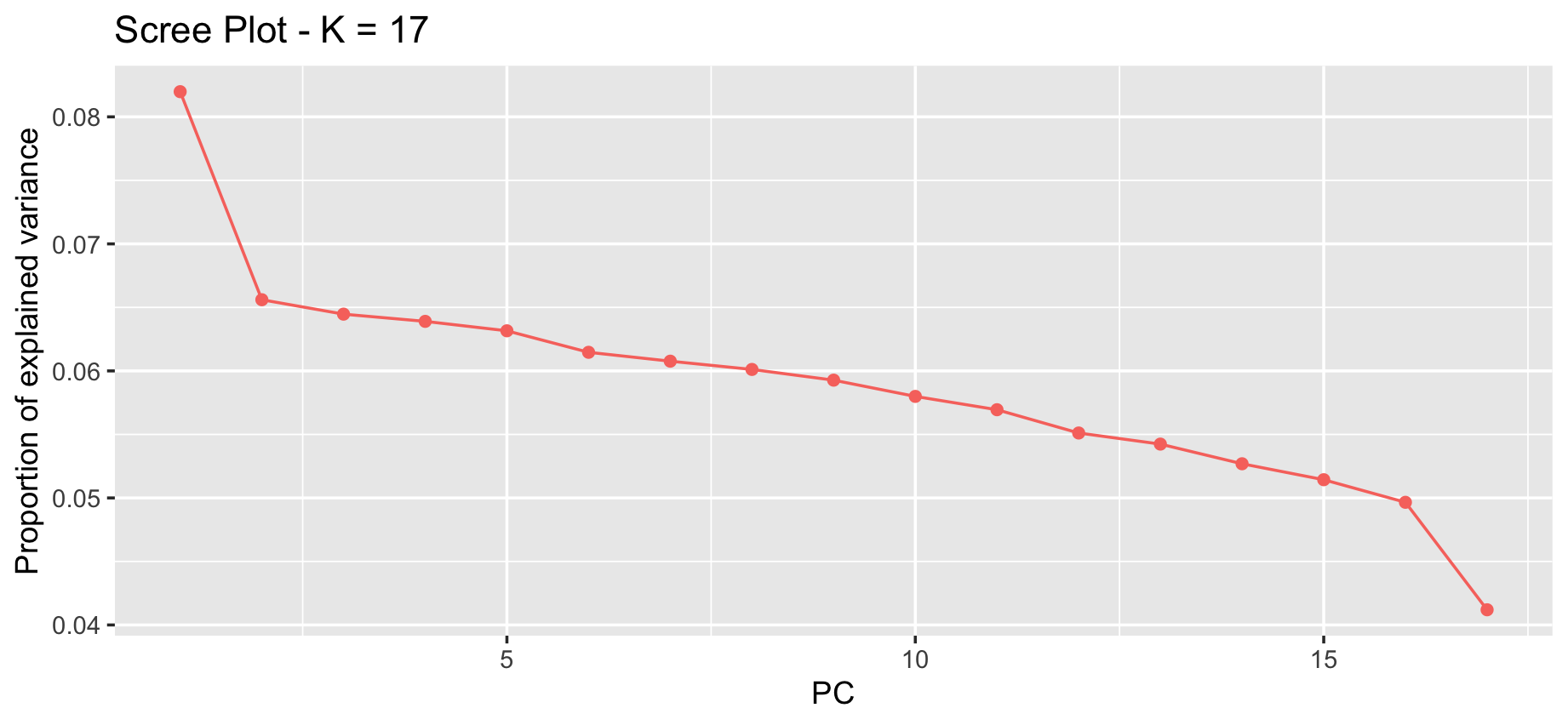
**

**Figure 3.** Eigenvalues of the partial RDA.

**
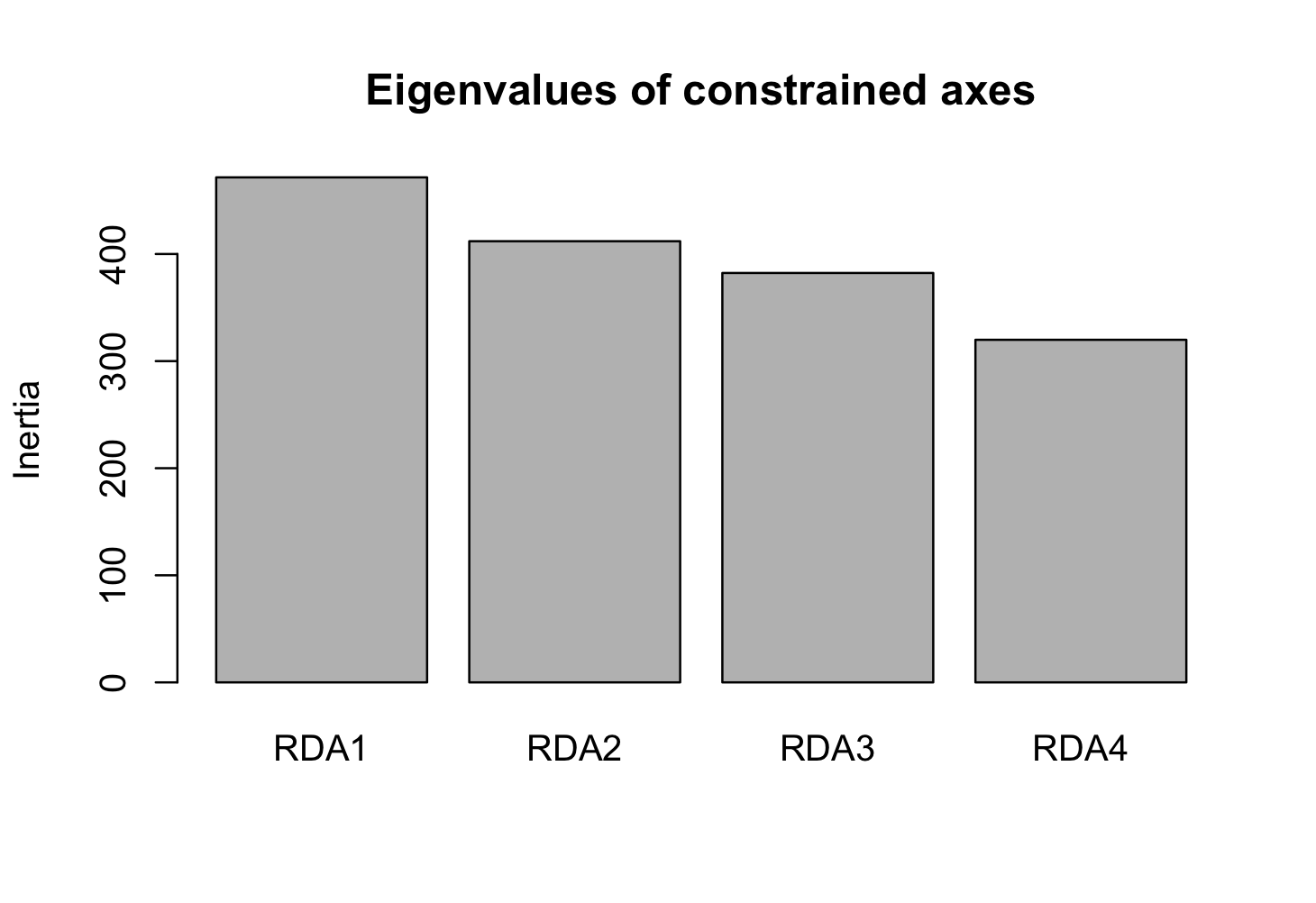
**

**Figure 4.** Eigenvalues of the simple RDA.

**
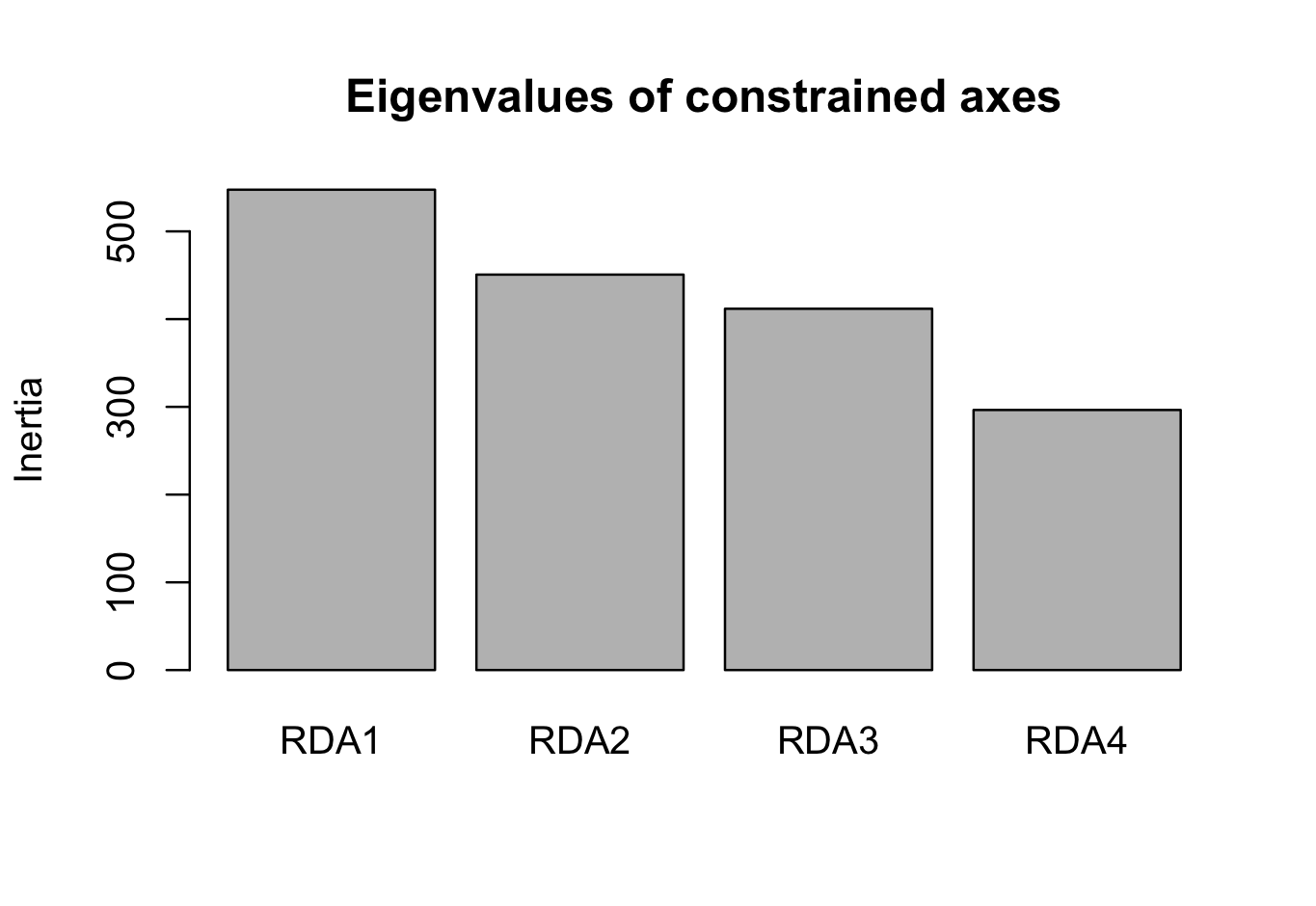
**

**Table 2.** List of enriched/depleted GO terms.

| **Ontology** | **Term** | **Frequency** | **Direction** |
| --- | --- | --- | --- |
| Cellular Component | membrane | 211 | Enriched |
| Molecular Function | binding | 135 | Enriched |
| Molecular Function | nucleic acid binding | 84 | Enriched |
| Biological Process | primary metabolic process | 75 | Enriched |
| Biological Process | DNA integration | 73 | Enriched |
| Molecular Function | ATP binding | 67 | Depleted |
| Molecular Function | transferase activity | 64 | Enriched |
| Molecular Function | catalytic activity | 57 | Enriched |
| Biological Process | macromolecule metabolic process | 54 | Enriched |
| Biological Process | cellular metabolic process | 53 | Enriched |
| Biological Process | regulation of biological process | 46 | Enriched |
| Cellular Component | cellular anatomical entity | 45 | Enriched |
| Biological Process | nucleic acid metabolic process | 40 | Enriched |
| Molecular Function | heterocyclic compound binding | 39 | Enriched |
| Molecular Function | organic cyclic compound binding | 39 | Enriched |
| Biological Process | DNA metabolic process | 37 | Enriched |
| Cellular Component | nucleus | 32 | Depleted |
| Biological Process | response to stimulus | 28 | Enriched |
| Biological Process | cellular process | 20 | Enriched |
| Biological Process | metabolic process | 20 | Enriched |
| Molecular Function | nucleotide binding | 20 | Enriched |
| Molecular Function | RNA-DNA hybrid ribonuclease activity | 20 | Enriched |
| Molecular Function | oxidoreductase activity | 18 | Depleted |
| Molecular Function | ion binding | 17 | Enriched |
| Cellular Component | intracellular organelle | 15 | Enriched |
| Cellular Component | membrane-bounded organelle | 14 | Enriched |
| Molecular Function | nucleotidyltransferase activity | 13 | Enriched |
| Molecular Function | iron ion binding | 9 | Depleted |
| Cellular Component | chloroplast | 8 | Depleted |
| Cellular Component | cytosol | 8 | Depleted |
| Biological Process | transport | 6 | Enriched |
| Molecular Function | aspartic-type endopeptidase activity | 6 | Enriched |
| Molecular Function | structural constituent of ribosome | 6 | Depleted |
| Biological Process | regulation of cellular process | 5 | Enriched |
| Biological Process | regulation of DNA-templated transcription | 5 | Enriched |
| Biological Process | carbohydrate metabolic process | 5 | Depleted |
| Biological Process | translation | 5 | Depleted |
| Molecular Function | protein folding chaperone | 5 | Enriched |
| Cellular Component | cullin-RING ubiquitin ligase complex | 4 | Enriched |
| Molecular Function | misfolded protein binding | 4 | Enriched |
| Molecular Function | DNA binding | 4 | Depleted |
| Biological Process | cell wall organization or biogenesis | 3 | Enriched |
| Biological Process | cellular component organization or biogenesis | 3 | Enriched |
| Biological Process | cellular response to unfolded protein | 3 | Enriched |
| Biological Process | cytidine to uridine editing | 3 | Enriched |
| Biological Process | mitochondrial mRNA modification | 3 | Enriched |
| Biological Process | pentacyclic triterpenoid biosynthetic process | 3 | Enriched |
| Biological Process | protein localization to membrane | 3 | Enriched |
| Biological Process | regulation of protein kinase activity | 3 | Enriched |
| Cellular Component | lipid droplet | 3 | Enriched |
| Cellular Component | protein kinase CK2 complex | 3 | Enriched |
| Molecular Function | beta-amyrin synthase activity | 3 | Enriched |
| Molecular Function | lanosterol synthase activity | 3 | Enriched |
| Molecular Function | protein kinase regulator activity | 3 | Enriched |
| Molecular Function | DNA-binding transcription factor activity | 3 | Depleted |
| Molecular Function | GTP binding | 3 | Depleted |
| Biological Process | chaperone-mediated protein complex assembly | 2 | Enriched |
| Biological Process | oxidation-dependent protein catabolic process | 2 | Enriched |
| Biological Process | positive regulation of mitochondrial translation | 2 | Enriched |
| Biological Process | regulation of mRNA processing | 2 | Enriched |
| Cellular Component | plasmodesma | 2 | Depleted |
| Molecular Function | [acyl-carrier-protein] S-malonyltransferase activity | 2 | Enriched |
| Molecular Function | lupeol synthase activity | 2 | Enriched |
| Molecular Function | mitochondrial ribosome binding | 2 | Enriched |
| Molecular Function | protein-disulfide reductase activity | 2 | Enriched |
| Molecular Function | translation activator activity | 2 | Enriched |
| Biological Process | chloride transport | 1 | Enriched |
| Biological Process | nuclear chromosome segregation | 1 | Enriched |
| Biological Process | pollen-pistil interaction | 1 | Enriched |
| Biological Process | tRNA guanine ribose methylation | 1 | Enriched |
| Cellular Component | autophagosome membrane | 1 | Enriched |
| Cellular Component | methylosome | 1 | Enriched |
| Cellular Component | ribonucleoside-diphosphate reductase complex | 1 | Enriched |
| Molecular Function | adenosylhomocysteine nucleosidase activity | 1 | Enriched |
| Molecular Function | guanidinoacetate N-methyltransferase activity | 1 | Enriched |
| Molecular Function | SUMO ligase activity | 1 | Enriched |
| Molecular Function | methyltransferase activity | 1 | Depleted |
| Biological Process | protein dephosphorylation | 0 | Depleted |
| Cellular Component | chloroplast envelope | 0 | Depleted |
| Cellular Component | chloroplast thylakoid membrane | 0 | Depleted |
| Cellular Component | endosome | 0 | Depleted |
| Molecular Function | antiporter activity | 0 | Depleted |

**Table 3.** List with all significant SNPs, identified by their chromosome and position inside it, and by which method/s have they been detected.

Check Table_3_suppl.csv

**Figure 5.** Map with K-means preferred clusters based on the allelic turnover pattern.


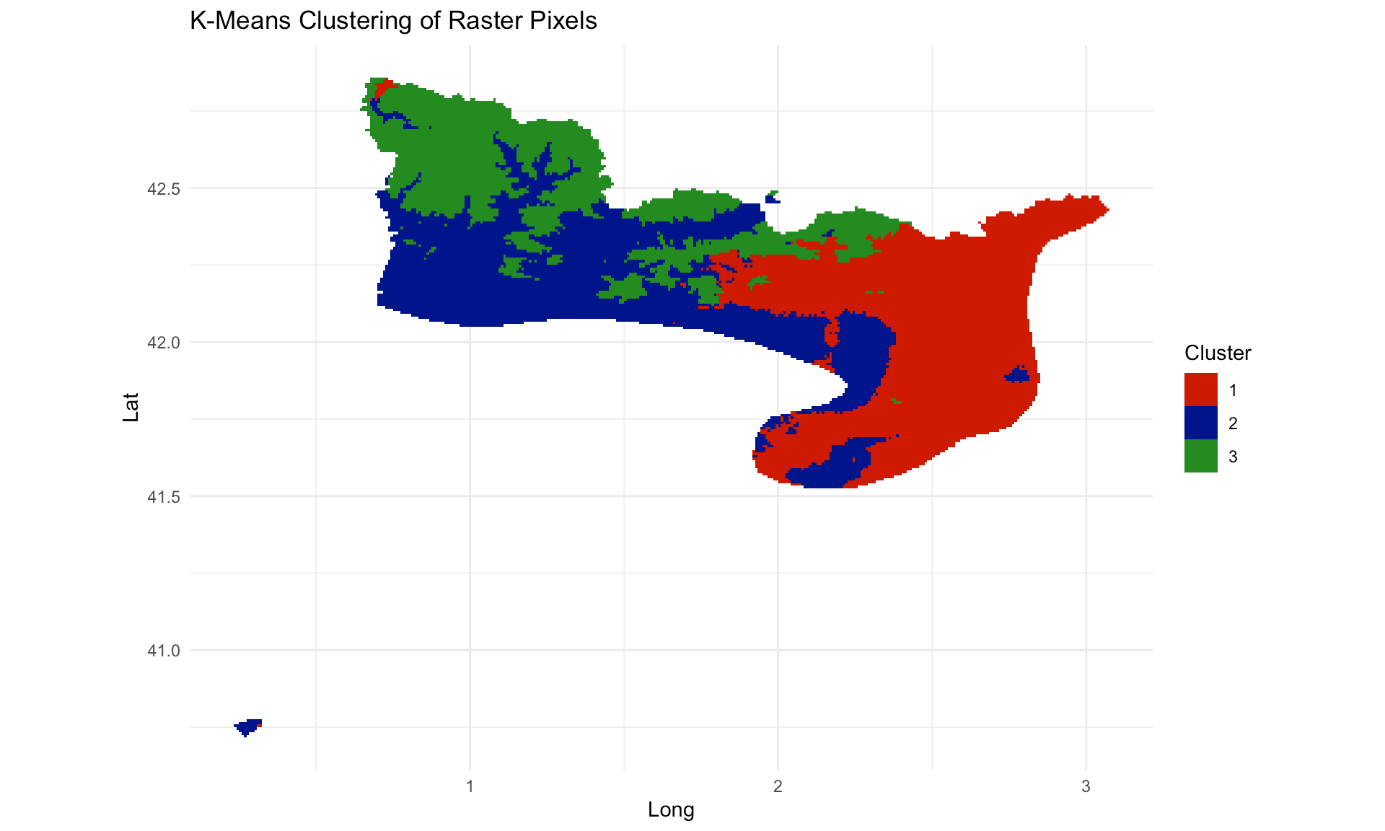


**Figure 6.** Offset maps 2061-2080 for all SSPs. The colour scale is equal for all maps.

**
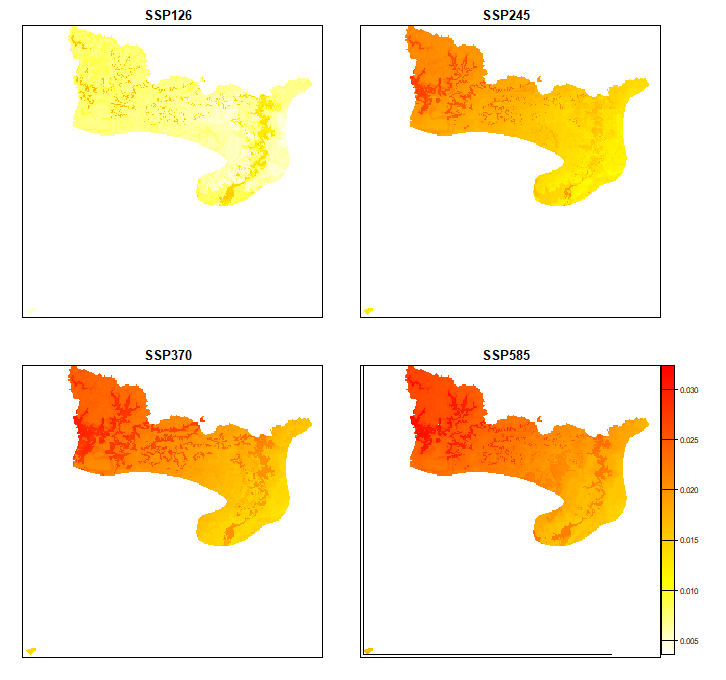
**

**Figure 7.** Linear regression between genetic offset (2061-2080) SSP585 and latitude (*p-value*=0.011; *R*-squared=0.3438).

**
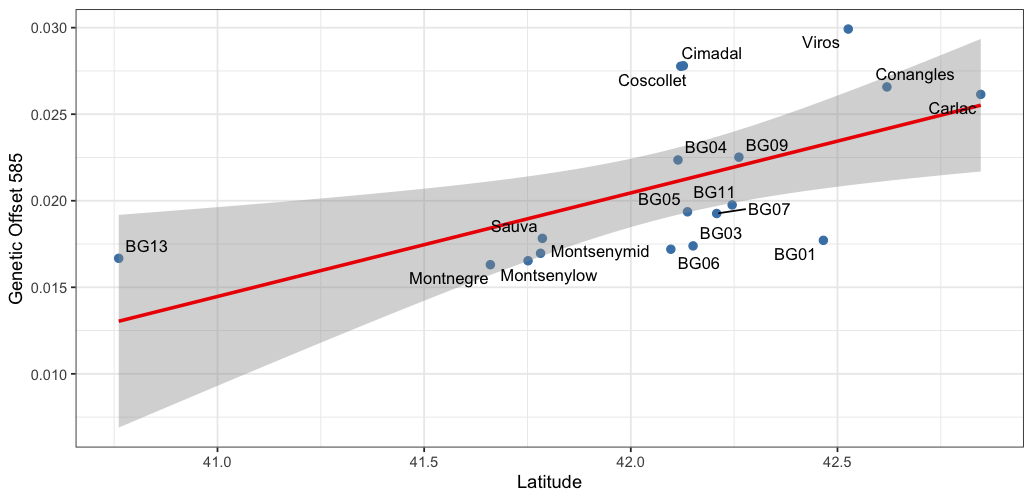
**

**Table 4.** Friedman+Conover post hoc test between different scenarios offset (2061-2080).

| Comparison | *p value* | Significance |  |
| --- | --- | --- | --- |
| SSP245 - SSP126 | <0.001 | *** |  |
| SSP370 - SSP126 | <0.001 | *** |  |
| SSP585 - SSP126 | <0.001 | *** |  |
| SSP370 - SSP245 | <0.001 | *** |  |
| SSP585 - SSP245 | <0.001 | *** |  |
| SSP585 - SSP370 | <0.001 | *** |  |
| Friedman test (χ² = 50.267, df = 3, p = 7.01E-11) | | | |
